# Digital Campaign to Promote COVID-19 Vaccination and Clinical Trial Participation Among Vietnamese Americans: Observational Engagement Study

**DOI:** 10.2196/90301

**Published:** 2026-10-15

**Authors:** Salik Tehami, Celine Nguyen, Janelle Hua, P Veronica Pham, Jannette Diep, Lauren Gilbert, Bich-May Nguyen

**Affiliations:** 1 University of Houston, 5055 Medical Circle, Houston, TX, 77204-6064, United States, 1 (713) 743-8514; 2 Vietnamese Culture and Science Association, Houston, TX, United States; 3 PIVOT, San Francisco, CA, United States; 4 Boat People SOS, Houston, TX, United States

**Keywords:** digital health communication, COVID-19, vietnamese Americans, health literacy, cultural competency, public health campaigns, mobile health, digital marketing metrics, language accessibility, community engagement

## Abstract

**Background:**

The COVID-19 pandemic necessitated innovative approaches to health communication, particularly for non–English-speaking communities. Vietnamese Americans, who report lower rates of English proficiency, faced unique challenges accessing facts and subsequent “infodemic” of rapidly spreading information.

**Objective:**

This study aims to evaluate the feasibility and engagement patterns of a community-engaged bilingual digital campaign delivered through Google’s Display & Video 360 (DV360) platform to Vietnamese Americans in Texas, comparing performance across multiple advertising formats.

**Methods:**

We implemented a 2-phase digital campaign on (DV360): static display advertisements (Phase 1: February 15-March 31, 2022) and audiovisual content (Phase 2: May 26-June 30, 2022). With community-based organizations (CBOs), 6 static displays, 2 videos, and 2 audio advertisements were developed in English and Vietnamese using focus group review and back-translation. Advertisement placements were constrained to a Texas Vietnamese audience segment. Total budget was US $9,472.54. Performance was evaluated using impressions, click-through rate (CTR), cost metrics, cost per click (CPC), cost per thousand impressions (CPM), and cost per view (CPV), with Wilson 95% CI, and two-proportion z-tests for substantive comparisons.

**Results:**

The campaign generated 4,974,357 impressions, reaching approximately 1.4 million unique users in Phase 1, and 105,000 in Phase 2. Vietnamese-language display advertisements achieved a higher CTR than English-language advertisements (0.105% vs 0.046%; *χ*_1_²=480.6; *P*<.001; risk ratio 2.29). Mobile placements outperformed desktop within each language (Vietnamese mobile 0.156% vs desktop 0.045%; English mobile 0.064% vs desktop 0.019%). Audio CTR was 1.911% in May but fell to 0.119% in June on small denominators (≈1800 impressions; 5 clicks). Impressions concentrated in expected Texas metropolitan areas (Houston 24.4%, Dallas 19.9%, Austin 7.9%). Phase 2 Google Analytics Behavior Flow showed 55.2% of language-distinguished landings were on Vietnamese pages (*χ*²_1_=6.7; *P*=.01). Cost efficiency was favorable (CPC US $2.35 and CPM US $1.45).

**Conclusions:**

A community-engaged bilingual digital campaign produced measurable in-language engagement among Vietnamese American audiences in Texas. Like other COVID-19 outreach campaigns directed at historically marginalized groups, researchers worked closely with CBOs, paying particular attention to messaging, language translation, and graphic representation in display, audio, and video advertisements to reach the population of interest. What differed from other campaigns was the use of DV360 allowing dissemination across multiple platforms and devices. Results are limited to engagement because direct impact on COVID-19 immunization or participation in clinical trials was not measured. Future directions can explore more clinically significant outcomes, in-language radio advertising, and harnessing AI to develop and monitor public health campaigns.

## Introduction

The COVID-19 pandemic precipitated a global health crisis and an unprecedented “infodemic”—a surge of information, including false or misleading content, that complicated public health communication efforts [1]. This challenge was particularly acute for non-English–speaking communities in the United States, who faced unique barriers in accessing reliable health information. Asian Americans, specifically Vietnamese Americans, encountered additional obstacles due to language barriers, cultural differences, and heightened discrimination during the pandemic [2,3]. The Asian American population comprises approximately 25 million people and is characterized by high mobile-device adoption and significant within-group heterogeneity in language preference and digital access [3].

Recent studies have highlighted how the rapid spread of misinformation through social media and online platforms disproportionately affected Asian American communities. Chong et al [4] documented how COVID-19 misinformation circulated through subgroup-specific, in-language social media platforms including TheKingRadio and TrainMaicoUSA on YouTube (Google LLC) among Vietnamese Americans and linked this exposure to potential downstream effects on vaccine decision-making and health protective behaviors. These challenges were compounded for Vietnamese Americans, who report lower rates of English proficiency compared to other Asian American groups and were disproportionately impacted by COVID-19 [5-8].

Digital health technologies emerged as crucial tools for disseminating public health information during the pandemic. However, their effectiveness varied significantly based on cultural and linguistic adaptation. Platforms theoretically capable of democratizing access often fail in implementation due to gaps in digital literacy, culturally inappropriate interface design, and language exclusion [9]. Specifically within Asian American communities, 56.1% of surveyed Asian American respondents had limited English proficiency, and the study linked those language access constraints to low COVID-19 testing uptake, underscoring the need for multilingual outreach strategies [10]. Asian American–serving community-based organizations (CBOs) bridged gaps by delivering culturally and linguistically centered COVID-19 outreach—including in-language social media content, webinars, and community health worker programs—reaching tens of thousands of community members [11].

Digital marketing platforms offer opportunities to reach diverse populations, but the literature systematically evaluating their effectiveness for multilingual health campaigns across multiple formats remains limited. Reviews of public health digital-campaign evaluation describe click-through rate (CTR) as a commonly used proximal engagement metric while emphasizing that values vary widely across platforms and do not by themselves establish behavior change [12]. Paid social-media health advertisements have had higher CTR than banner or display advertisements, with banner or display median CTRs of approximately 0.08%‐0.13% across analyses by the HEAL (Helping to End Addiction Long-Term) Communities Study [13]. Adjacent multilingual campaigns by the National Heart Foundation of Australia have reported higher CTRs in translated-video and search-advertisement contexts — translated Vietnamese and Mandarin Facebook (Meta Platforms, Inc) video outreach yielded higher CTR than English-language posts (0.77% vs 0.62%) [14], and multilingual Google AdWords recruitment to a health research website found CTR varied substantially by language [15].

Recent work has examined digital outreach to broader racial and ethnic populations: analyzing COVID-19 testing and vaccine Twitter (Twitter Inc.; subsequently rebranded as X) posts from Houston government agencies, elected officials, and CBOs found that government agencies were most likely to address access, fear, and language barriers [16]; a social media microinfluencer flu vaccine campaign among African American and Hispanic audiences reported 69,495 engagements and improvements in flu-vaccine beliefs and community-attitude measures [17]. The Stanford Center for Asian Health Research and Education (CARE) used a 10-week social media campaign across Facebook, Instagram (Meta Platforms, Inc), and Twitter to increase the percentage of Asian participants in the Stanford Research Registry from 525 to 1871 individuals, providing one of the few recent quantitative benchmarks for digital outreach to Asian American populations [18]. Park et al [19] have explored digital recruitment strategies for Asian Americans and Pacific Islanders more broadly. However, few studies have systematically evaluated different digital formats—static, video, and audio—specifically in reaching Vietnamese American audiences during public health emergencies, and the format-versus-other-design-parameters question (language, device, and placement) has received little empirical attention.

In response to the COVID-19 pandemic, the Texas Community-Engagement Research Alliance against COVID-19 (CEAL) Consortium formed in August 2020 [20]. As part of the Consortium, this study describes a community-engaged digital campaign directed at Vietnamese Americans in Texas to promote COVID-19 vaccinations and clinical trial participation. Using an online marketing platform, we evaluate the engagement and placement characteristics of multiple digital advertising formats in English and Vietnamese, providing observational insights into design parameters that govern engagement for linguistically diverse diaspora populations.

## Methods

### Study Design

This observational study evaluated a bilingual digital health campaign focused on Vietnamese Americans in Texas implemented between February 15 and June 30, 2022. The campaign was structured in 2 distinct phases: an initial display advertisement phase from February 15 to March 31, 2022, followed by an audiovisual content phase from May 26 to June 30, 2022. The total campaign budget was US $9472.54, with US $5879.42 allocated to display advertisements and US $3593.12 to YouTube, connected television, and digital-radio audio content. CBO partnerships with PIVOT and Boat People SOS-Houston (BPSOSH) supported community engagement and campaign development. The CONSORT-EHEALTH (Consolidated Standards of Reporting Trials of Electronic and Mobile Health Applications and Online Telehealth) checklist was completed and uploaded as Checklist 1 [21].

### Campaign Design

The campaign featured 6 health messages identified through a community-engaged approach led by PIVOT in collaboration with the academic research team: COVID-19 vaccine safety, family protection, and pregnancy safety, and 3 messages encouraging clinical trial participation. Message themes were informed by existing research on Asian American vaccination messaging and clinical trial recruitment, and by recent work on cultural adaptation of digital health interventions, emphasizing that effective adaptations extend beyond translation to include alignment with users’ contextual socioeconomic and platform realities [22-24].

PIVOT developed an initial set of English-language messages, which were translated into Vietnamese by Vietnamese-speaking PIVOT staff. Vietnamese advertisement copy was reviewed and refined through a back-translation procedure: a PIVOT reviewer translated each Vietnamese version back into English, and BPSOSH compared the back-translations with the originals to confirm semantic equivalence and cultural appropriateness (File S1 [Edited Advertisements for Back-Translation]; File S2 [Edited Advertisements with Vietnamese Translation] in Multimedia Appendix 1).

BPSOSH convened focus groups of Vietnamese American community members to review the draft advertisements as described previously [25]. Each advertisement received structured positive and critical feedback. Focus-group insights were operationalized into specific design revisions documented in Table S3 in Multimedia Appendix 1 (focus-group feedback → advertisement evolution). Specific examples include the following:

- Figure 1 (vaccine safety): the original copy “COVID-19 vaccines are unlikely to cause long-term side effects because vaccine ingredients are cleared from the body quickly” was flagged as confusing and too long. The revised copy was simplified to “COVID-19 vaccines are safe” / “Vắc xin COVID-19 là an toàn.”

**Figure 1.**
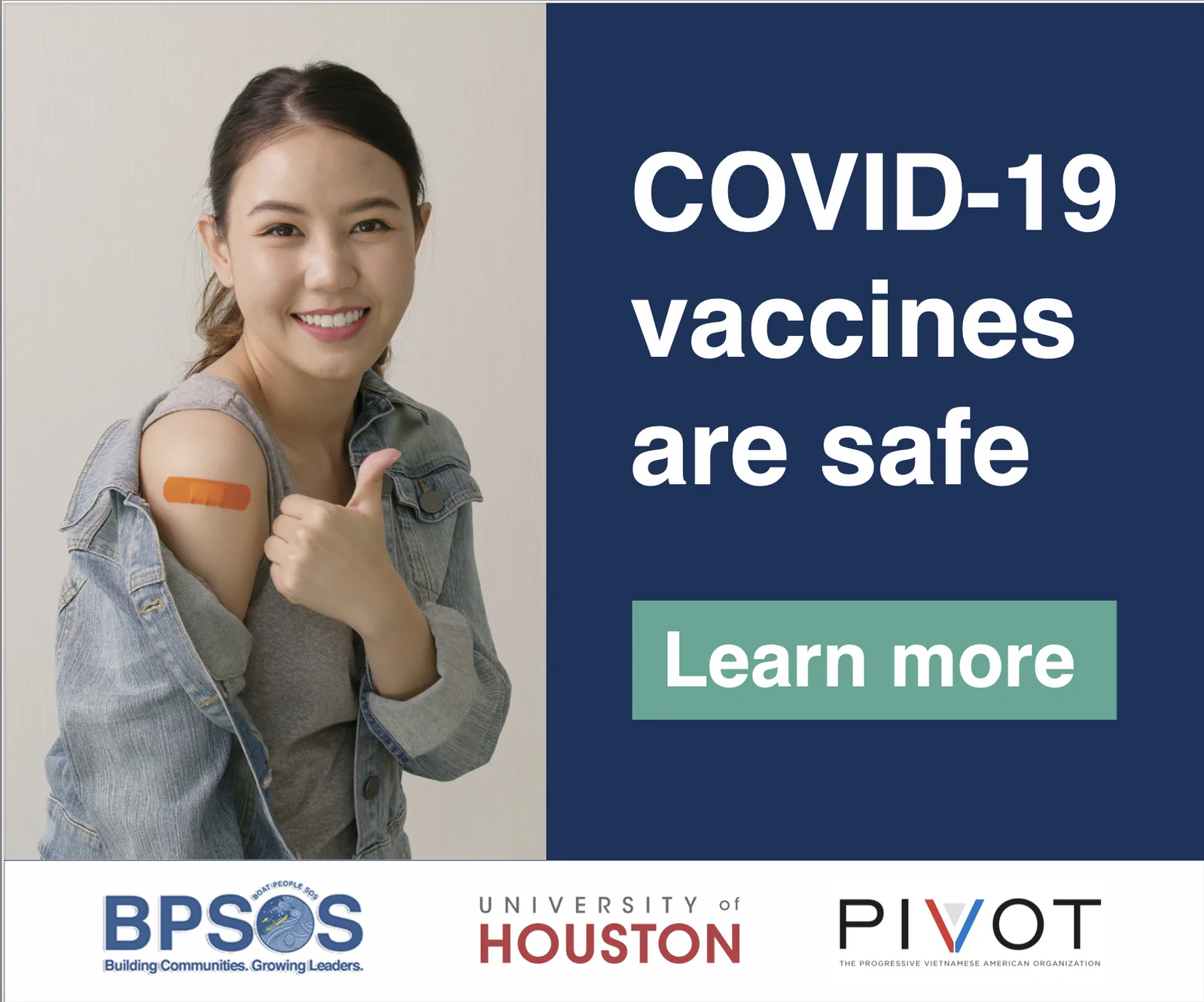
Example of static advertisements used in the first phase of the campaign. Message: COVID-19 vaccines are safe (Source: photo purchased from Shutterstock).

- Figure 2 (family protection): focus-group participants requested imagery depicting multiple generations rather than a single adult. The final advertisement used a multigenerational family photo paired with the copy “Getting a COVID-19 vaccine can help protect you from severe illness from COVID-19.”

**Figure 2.**
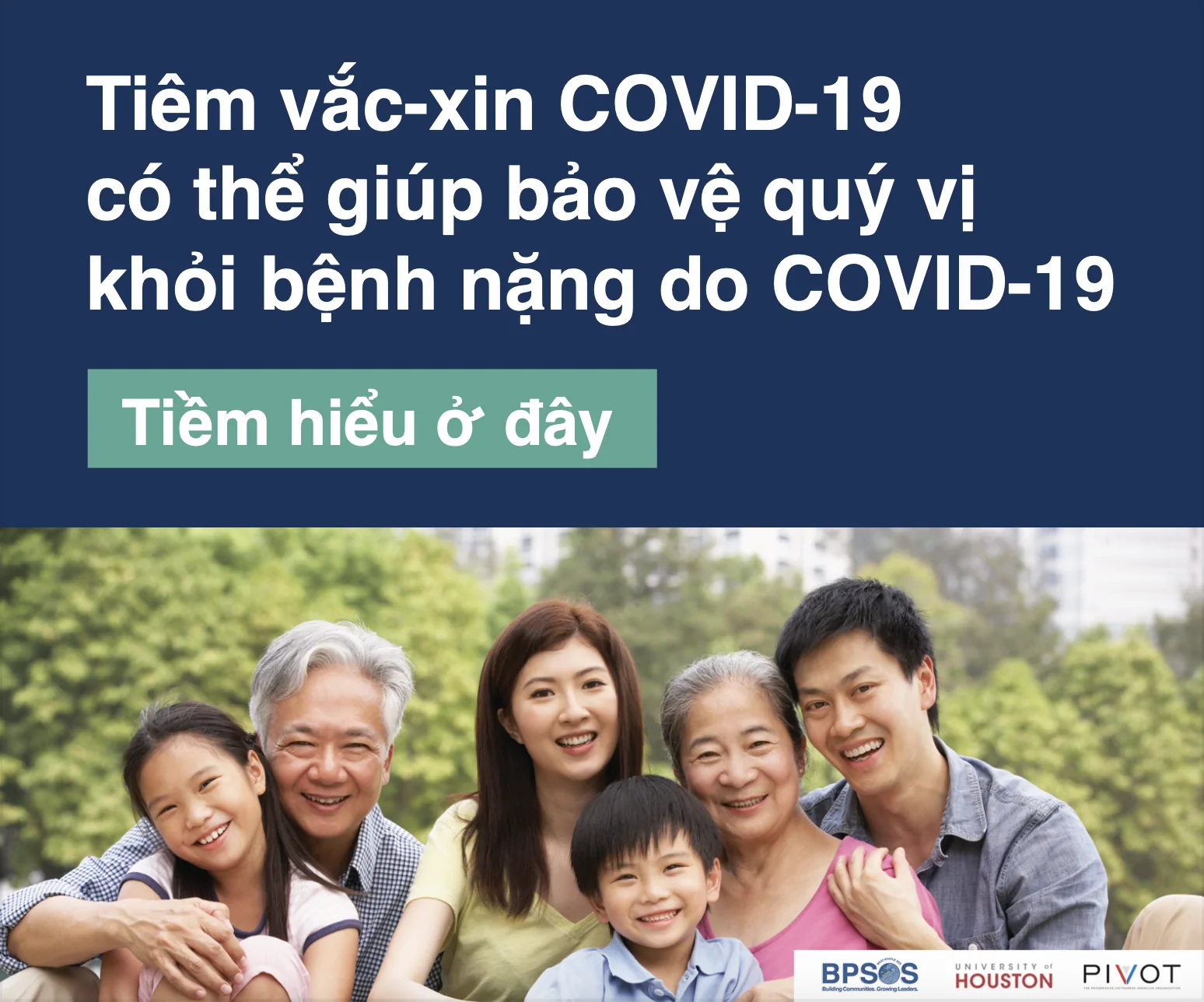
Example of static advertisements used in the first phase of the campaign. Message: Tiêm vắc-xin COVID-19 có thể giúp bảo vệ quý vị khỏi bệnh nặng do COVID-19. English translation: Getting a COVID-19 vaccine can help protect you from severe illness from COVID-19 (Source: photo purchased from Shutterstock).

- Figure 3 (pregnancy): participants reported the original copy framed pregnancy as a vaccine side effect; the revised wording—“COVID-19 vaccination before, during, or after pregnancy is safe”—placed safety in the foreground, and the image was changed to depict a pregnant person’s face and upper body rather than an isolated gravid abdomen.

**Figure 3.**
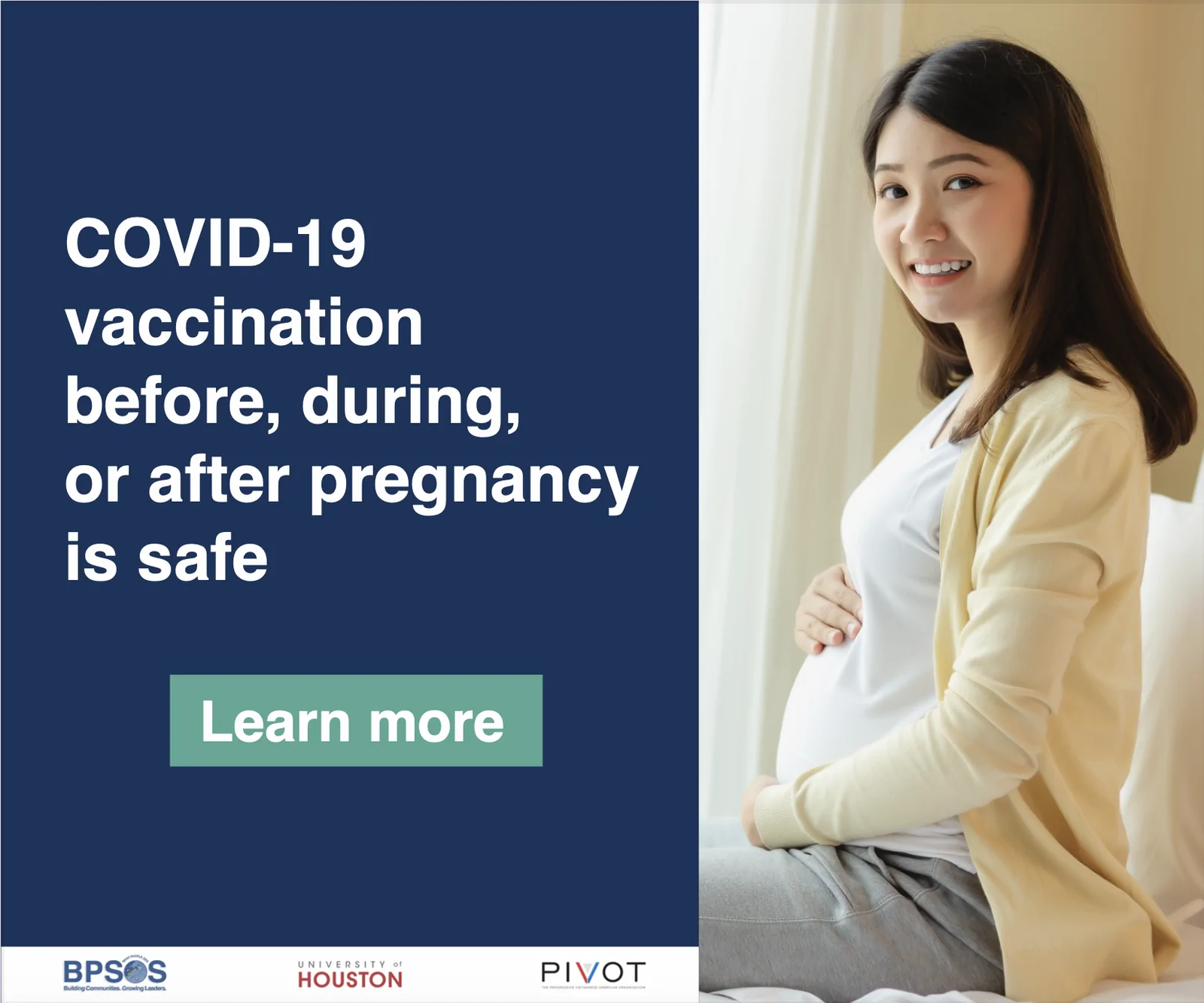
Example of static advertisements used in the first phase of the campaign. Message: COVID-19 vaccination before, during, or after pregnancy is safe (Source: photo purchased from Shutterstock).

- Figure 4 (clinical trials): participants reported that the term “trial” connoted risk and unknown outcomes, while “study” implied broader benefit. Vietnamese copy was reworded as “Tham gia nghiên cứu COVID-19” (participate in COVID-19 research) rather than the literal translation of “trials.”

**Figure 4.**
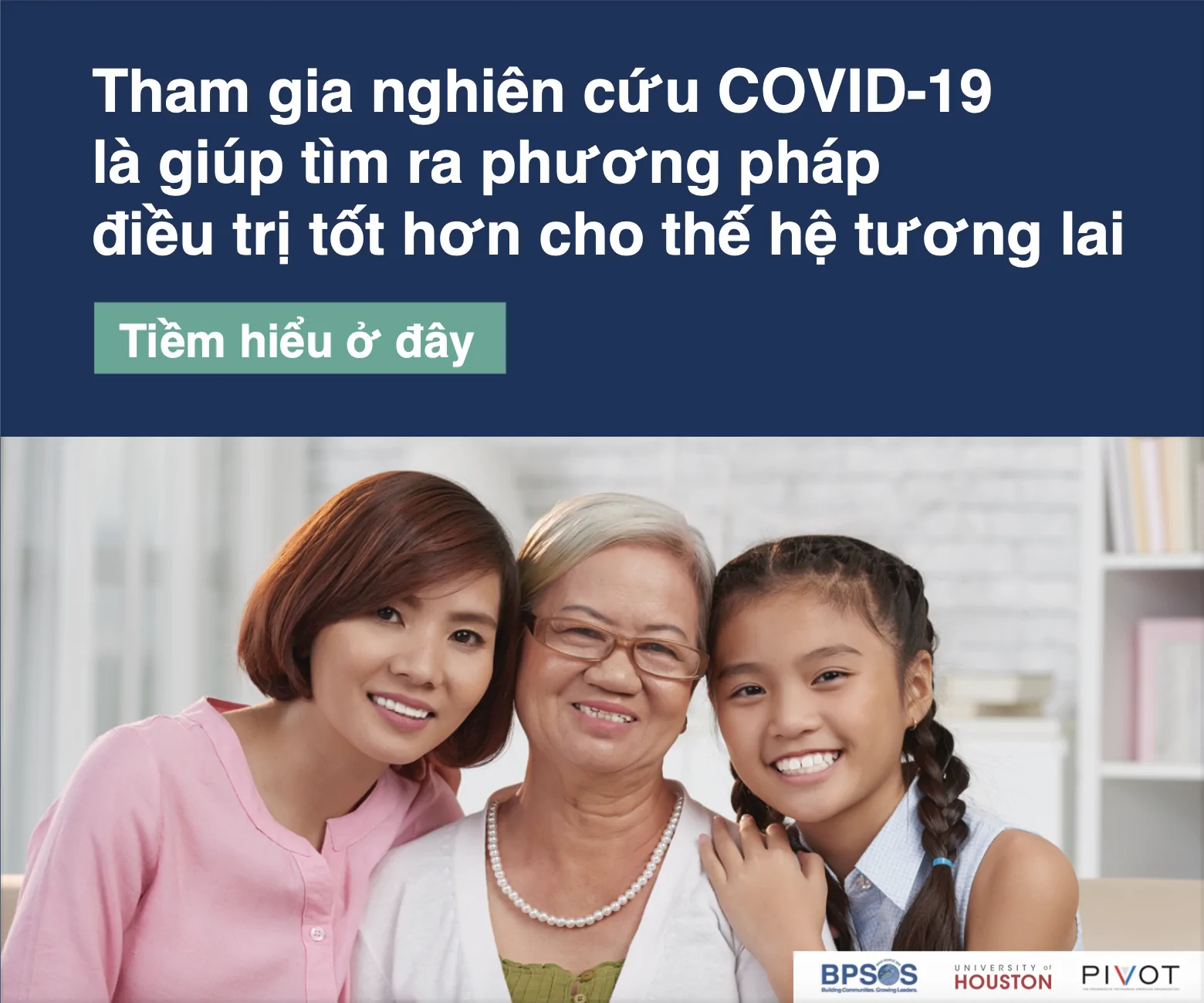
Example of static advertisements used in the first phase of the campaign. Message: Tham gia nghiên cứu COVID-19 là giúp tìm ra phương pháp điều trị tốt hơn cho thế hệ tương lai. English translation: Participating in COVID-19 research studies helps find better treatments for future generations (Source: photo purchased from Shutterstock).

- Figures 5 and 6 (clinical trials and framing): participants requested more personal framing and clearer indication that the trials were COVID-19–specific. Both advertisements were revised to lead with “Your participation”/“Quý vị tham gia” and to specify COVID-19.

**Figure 5.**
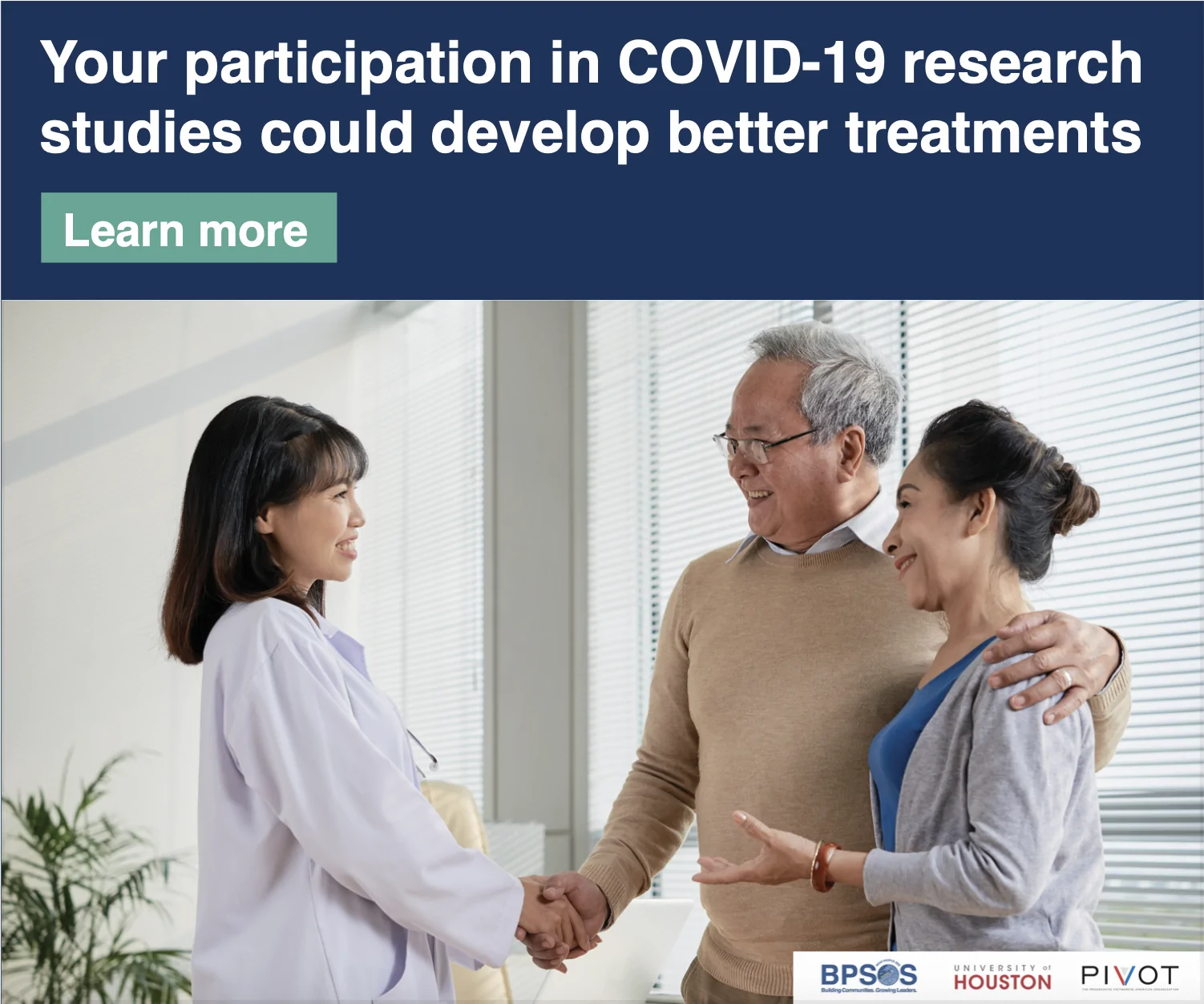
Example of static advertisements used in the first phase of the campaign. Message: your participation in COVID-19 research studies could develop better treatments (Source: photo purchased from Shutterstock).

**Figure 6.**
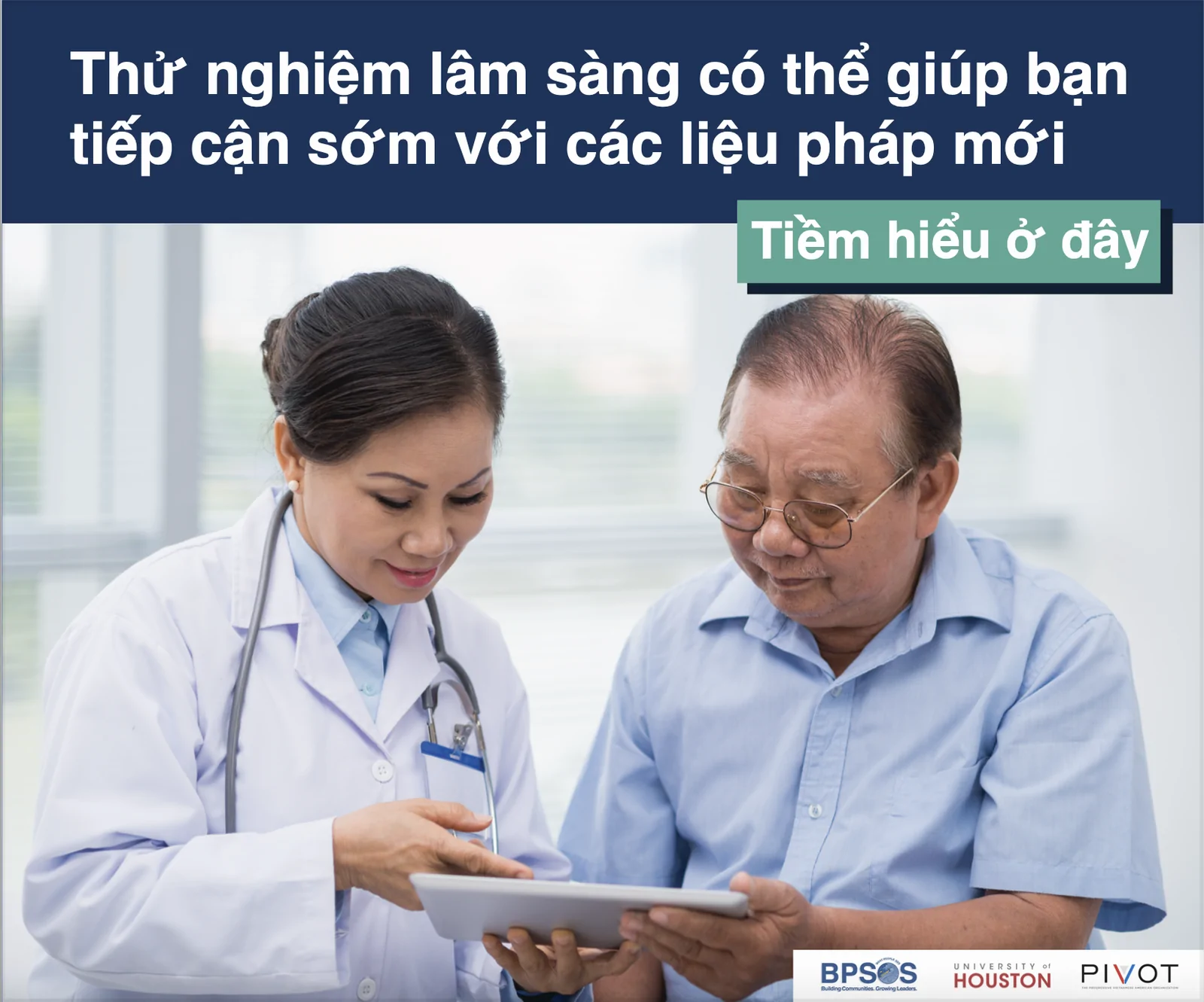
Example of static advertisements used in the first phase of the campaign. Message: Thử nghiệm lâm sàng có thể giúp bạn tiếp cận sớm với các liệu pháp mới. English translation: Clinical trials may give you early access to new therapies (Source: photo purchased from Shutterstock).

Design elements specifically tailored for the Vietnamese American audience included (1) imagery depicting people who phenotypically appeared Vietnamese, East Asian, or Southeast Asian, sourced from stock libraries using search terms beginning with “Vietnamese” and broadened to “Asian” when supply was limited; (2) family- and intergenerational framing in images, responding to focus-group feedback emphasizing family-oriented appeals; (3) Vietnamese register and idiom choices reviewed by community-partner staff; (4) use of the term “nghiên cứu” (research) rather than “thử nghiệm” (trial) where focus-group feedback indicated this was less threatening; and (5) inclusion of PIVOT and BPSOSH logos to anchor the messages in trusted community-organizational sources. Messaging and translation focused on using common words with short, simple messages. Design elements common to both language versions were sans-serif typefaces with high-contrast backgrounds for readability and inclusion of the academic and community-partner logos. Selected images included people who looked happy or in medical settings.

All 6 static-display advertisements were produced in 7 dimensions (120×600, 160×600, 180×150, 300×250, 300×600, 468×60, 728×90) to permit placement across desktop and mobile inventory. Audiovisual advertisements ranged from 1:14 to 2:05 in length, each in English and Vietnamese, and are included in Multimedia Appendices 2-5. Audio versions used the audio tracks of the video advertisements, served as stand-alone audio creatives via digital-radio inventory. A frequency cap of 12 impressions per unique user was applied to limit audience fatigue. The revised advertisements were deployed at the February 15, 2022, campaign launch without a separate pilot phase or A/B test; the focus-group review with BPSOSH constituted the only formal prelaunch validation step.

### Digital Platform Implementation

The campaign was placed through Google’s Display & Video 360 platform (DV360), which delivers across more than 95 advertising exchanges and aggregates inventory from content websites, in-app placements, digital-radio networks (including audio versions of broadcast content), connected television apps, and YouTube. Unlike platform-native social media campaigns, DV360 does not place advertisements on Facebook, Instagram, or TikTok (ByteDance). Placement of each impression was determined by DV360’s automated bidder using a combination of explicit targeting parameters set by the campaign team and algorithmic optimization for the configured performance objective.

Explicit targeting parameters were:

- Geography: users located in the state of Texas.
- Language signals**:** device or session language preference set to English (for English creatives) or Vietnamese (for Vietnamese creatives).
- Audience segment (Phase 2 only): in the video, radio, or YouTube phase (May 26 – June 30, 2022), placements were additionally constrained to a “Texas Vietnamese” audience segment of approximately 300,000 users defined by DV360’s language-and-locale signals.
- Contextual signals**:** PIVOT staff with prior experience in Vietnamese American digital outreach assembled a list of topic- and language-relevant contextual keywords used by DV360’s contextual-targeting layer to identify content pages and applications likely to be consumed by Vietnamese American users in Texas. Keywords spanned 6 categories: (1) Vietnamese-language news and media, (2) family- and parenting-related content, (3) education and learning content, (4) Vietnamese-language entertainment, (5) general COVID-19 and health-information categories, and (6) clinical-research and trial-related terms. The list was assembled prior to launch from prior community-engagement campaigns by the same CBO partner (Table S4 in Multimedia Appendix 1. Keyword effectiveness was monitored indirectly through the platform’s placement reports; the top-app and top-website distributions for each language arm (Tables S5 and S6 in Multimedia Appendix 1) provide direct evidence of where contextual signals routed impressions. The DV360 bidder integrated geographic constraint, language signal, frequency cap, audience-segment overlay (Phase 2), and keyword signal algorithmically.
- Frequency cap: 12 impressions per unique user across the campaign period.
- Creative rotation: DV360’s auto-optimization was enabled, with the platform giving heavier delivery to better-performing creatives.

### Data Collection and Metrics

Performance data were collected through DV360 and Google Analytics (GA) platforms using standardized digital marketing metrics. Key engagement metrics included impressions (the number of times advertisements were displayed to a user), reach (the total number of viewers), clicks (user interactions with advertisements), and CTR (calculated as clicks divided by impressions). For video content delivered through YouTube, connected television, and YouTube-engine partners, impressions were counted using Google’s TrueView standard, which records a view only when a viewer watches at least 80% of the advertisement. Digital radio advertisements were counted as impressions when the audio played for any duration. Financial performance was tracked through cost per click (CPC), cost per thousand impressions (CPM), and cost per view (CPV) for video content.

Website engagement was monitored using several metrics, including traffic sources, bounce rates, average session duration, and pages per session. Language preference patterns were tracked to understand the effectiveness of bilingual content delivery. The platform also provided detailed information about device types and the geographic distribution of the audience. Individual users and research team members were not blinded to the intervention or analysis.

### Theoretical Framework

The campaign was based on a few theories to promote COVID-19 vaccination and clinical trial participation. First, the Health Belief Model (HBM), which focuses on perceived susceptibility, perceived severity, perceived benefits, perceived barriers, cues to action, and self-efficacy [26]. Advertisements highlighted disease risk, vaccine effectiveness, and vaccine safety. Additionally, the Protection Motivation Theory (PMT) posits that people’s health-related behaviors are a function of 6 components: which focuses on perceived severity, perceived vulnerability, outcome efficacy, self-efficacy, response costs, and maladaptive rewards [27]. Campaign messages emphasized disease consequences and confidence in vaccine protection. Furthermore, the Diffusion of Innovations theory, which concentrates on innovation characteristics and opinion leaders [28]. The advertisements about participating in therapeutic trials highlighted developing new treatments and display advertisements linked to information from a trusted source that could be shared.

We organized the analysis around the Elaboration Likelihood Model (ELM) of persuasion [29]. ELM distinguishes central-route processing—effortful evaluation of message content—from peripheral-route processing, which relies on format affordances, source cues, and ease of processing. For diaspora audiences with varying English proficiency, peripheral-route channels (in-language audio, mobile-optimized in-language display) may produce higher engagement than central-route channels (long-form English text) by lowering cognitive load and matching mobile-first media-consumption defaults [3]. Format, language, device, and engagement are therefore interpreted as joint inputs to cognitive load rather than independent design choices.

We treat this as a framing expectation rather than a tested hypothesis; the cell-level CTRs reported in Table 1 are interpreted as consistent or inconsistent with ELM-route predictions, not as confirming them. Contemporary social-media-effects models additionally argue that engagement, not awareness, is the catalyst for behavior change in digital campaigns, framing our engagement metrics as inputs to a nonlinear model rather than proxies for behavioral outcomes [30].

**Table 1.** Campaign performance metrics by format, language, and topic (February-June 2022^a^).

Format	Language	Topic	Impressions (N=4,974,357)	Clicks (N=3136)	CTR^b^ (overall pooled rate 0.063%), %	95% CI
Display	English	Vaccination	1,124,659	541	0.048	0.044%‐0.052%
Display	English	Clinical trial	1,321,368	581	0.044	0.041%‐0.048%
Display	Vietnamese	Vaccination	674,562	766	0.114	0.106%‐0.122%
Display	Vietnamese	Clinical trial	858,445	844	0.098	0.092%‐0.105%
Video	English	Vaccination	247,387	70	0.028	0.023%‐0.036%
Video	English	Clinical trial	244,766	81	0.033	0.027%‐0.041%
Video	Vietnamese	Vaccination	330,579	167	0.051	0.043%‐0.059%
Video	Vietnamese	Clinical trial	170,757	81	0.048	0.038%‐0.059%
Audio	English	Vaccination	262	1	0.382	0.067%‐2.130%
Audio	English	Clinical trial	297	2	0.673	0.185%‐2.422%
Audio	Vietnamese	Vaccination	633	1	0.158	0.028%‐0.890%
Audio	Vietnamese	Clinical trial	642	1	0.156%	0.027%‐0.877%

a CTRs computed from monthly per-creative impressions and click-through rates exported from Google’s Display and Video 360 advertising platform (DV360). Wilson 95% CI are reported for each cell because click totals in audio cells are small. Inferential comparisons: Vietnamese-language display achieved a higher CTR than English-language display (*χ*²_1_=480.6; *P* <.001; risk ratio 2.29, 95% CI 2.12–2.47). Display as a class achieved a higher CTR than video (*χ*²_1_=102.6; *P*<.001). Audio CTR (0.273% across 1834 impressions and 5 observed clicks) was statistically higher than display CTR (*χ*²_1_=11.1; *P*=.001) but rests on a small click count and is sensitive to small variations in count. Audio impressions reflect digital-radio inventory served in May–June 2022; an additional 304,424 unclassified impressions in June 2022 are reported under cross-platform engagement.

b CTR: click-through rate.

### Statistical Analysis

Performance metrics—impressions, clicks, CTR, CPC, CPM, and CPV—were extracted from DV360 monthly per-creative exports and aggregated to the format×language×topic level (Table 1) and to the line-item level for device-specific analyses (Table 2). CTRs are reported with Wilson 95% CIs to account for the small denominators in the audio cells.

**Table 2.** Display CTR^a^ by language and device type (Phase 1: 15 February – 31 March 2022). Vietnamese mobile display achieved the highest nonaudio CTR observed in the campaign (Source: DV360^b^ line-item CTR export).

Language^c^	Device^d^	CTR^a^, %
English	Desktop	0.019
English	Mobile	0.064
Vietnamese	Desktop	0.045
Vietnamese	Mobile	0.156

a CTR: click-through-rate.

b DV360: Google’s Display & Video 360 advertising platform.

c Vietnamese outperformed English within each device.

d Mobile outperformed desktop within each language.

For the substantively important comparisons, we report two-proportion z-tests (equivalent to Pearson *χ*² on the 2×2 table of clicks and nonclicks) at the platform-aggregate level: (1) Vietnamese versus English display CTR; (2) display versus video CTR; and (3) audio versus display CTR. We treat these as descriptive comparisons of platform-level aggregates rather than tests of individual-level hypotheses because impressions are not independent user observations (the same user may receive multiple impressions under the frequency cap of 12) and because format-level comparisons are confounded with phase, audience-segment targeting, and the platform’s automated bidder. Effect sizes are reported as risk ratios alongside *P* values.

We did not perform parametric tests (*t* tests or ANOVA) on monthly CTR observations within format. With 2 to 3 monthly observations per medium and confounding between format and phase, parametric inference at the medium-month level is not adequately powered. The reported month-level CTR variation is presented descriptively. Website-engagement metrics from GA—sessions, language distribution, bounce rate, session duration, and pages per session—are tabulated by campaign phase and a precampaign baseline window. Phase 2 landing-page distribution by language was inferred from GA Behavior Flow reporting because each advertisement pointed to a specific language–distinguished landing URL.

### Ethical Considerations

This research was approved by the University of Houston Institutional Review Board (STUDY00003046) on July 21, 2021. All campaign materials and data collection procedures followed Google’s advertising policies and health information guidelines.

## Results

### Overall Campaign Reach

The digital health campaign achieved substantial audience exposure, generating 4,974,357 classifiable impressions across all formats over a 19-week period plus 304,424 unclassified June 2022 impressions reported separately under cross-platform engagement (Table 1). The campaign reached approximately 1.4 million unique users during the display phase (frequency 2.8 advertisement views per person) and approximately 105,000 unique users during the audiovisual phase (File S9 [DV360 reach summary] in Multimedia Appendix 1). Platform-reported frequency for the audiovisual phase (3.06 average views per person) uses Google’s TrueView view-based counting and cannot be multiplied against reach to reproduce served impressions; reach and impression counts are reported separately for this reason. Impressions reflect advertisement delivery within geographic targeting and platform-side language, contextual, and audience-segment signals; the proportion of impressions served to Vietnamese American users is not directly measurable from platform-side reporting. Table 1 summarizes campaign performance by format, language, and topic.

### Performance of Static Display Advertisements

English-language static advertisements achieved 2,446,027 impressions; Vietnamese-language counterparts generated 1,533,007 impressions (Table 1). Performance varied by display dimension and placement; per-dimension impression counts are reported in Table S9 in Multimedia Appendix 1.

### Performance of Audiovisual Advertisements

Video advertisement impressions ranged from 170,757 to 330,579 across the 4 language-topic cells. The Vietnamese-language vaccination video achieved the highest engagement (330,579 impressions; CTR 0.051%), followed by the English vaccination video (247,387 impressions; CTR 0.028%), English clinical trial video (244,766 impressions; CTR 0.033%), and Vietnamese clinical trial video (170,757 impressions; CTR 0.048%; Table 1).

The audio component reached a smaller audience. Audio advertisements about COVID-19 vaccination narrated in English received 262 impressions and Vietnamese 633 impressions; clinical trial audio impressions were 297 (English) and 642 (Vietnamese). Across all 4 audio cells, 5 clicks were observed on 1834 impressions (Table 1).

### Platform and Device Engagement

Analysis of device-attributed impressions for the 4 video creatives revealed that mobile phones dominated content delivery, generating 608,428 impressions (608,428/993,489, 61.2% of device-attributed impressions; Table 3). Desktop computers followed with 338,370 impressions (338,370/993,489, 34.1%), while tablets and connected-television devices accounted for 46,557 and 134 impressions, respectively (Table 3). The 993,489 total in Table 3 comprises the 4 video creatives exactly (Table 1 videocells: 247,387+244,766+330,579+170,757), delivered across web video, YouTube, and connected-television inventory. Audio impressions (n=1834) and static-display impressions are not device-segmented in DV360’s per-creative export and are therefore excluded from Table 3.

**Table 3.** Device-attributed impressions for video creatives by device type and language (delivered across web video, YouTube, and connected-TV inventory; audio and static-display impressions not device-attributed).

Device type	Total impressions (N=993,489)	English impressions (N=695,443)	Vietnamese impressions (N=298,046)	% of device-attributed reach (100%)
Mobile phone	608,428	425,900	182,528	61.20
Desktop	338,370	236,859	101,511	34.10
Tablet	46,557	32,590	13,967	4.70
Connected TV	134	94	40	0.01

### Engagement and Cost Efficiency Metrics

The campaign’s overall CTR was 0.063%. Cost efficiency was evaluated using multiple metrics. The average CPC across the campaign was US $2.35, with individual advertisements ranging from US $0.03 to US $9.95 (Table 1). The CPM averaged US $1.45 (range US $0.70-US $5.12). The video content CPV averaged US $2.31, with minimal variation (US $2.19-US $2.51).

### Temporal Patterns and Inferential Comparisons

Aggregated monthly CTRs differed substantially within format. Standard display CTRs were stable at 0.066%‐0.074% across February and March 2022 and 0.057% in June. Video CTR was stable at 0.040% in both May and June. Audio CTR was 1.911% in May 2022, falling to 0.119% in June—a 16-fold drop on a small impression base. The May audio CTR of 1.911% reflects 3 clicks on the 157 audio impressions served that month, all on English-language creatives (1 vaccination, 2 clinical trial); the June rate of 0.119% reflects 2 clicks on 1677 impressions, both on Vietnamese-language creatives (1 vaccination, 1 clinical trial). The monthly and cell-level accountings therefore reconcile to the same 5 total clicks on 1834 audio impressions reported in Table 1. The rate did not replicate in June, and we interpret the audio observation cautiously.

Two-proportion comparisons across format–language cells confirm 3 differences. First, Vietnamese display achieved a higher CTR than English display (*χ*_1_²=480.6; *P*<.001; risk ratio 2.29). In absolute terms, this corresponds to approximately 10.5 versus 4.6 clicks per 10,000 impressions—roughly 6 additional clicks per 10,000 impressions delivered. The large *χ*² value reflects the very large impression denominators rather than a large absolute difference; at campaign scale (1.5 million Vietnamese-language display impressions), the absolute difference nonetheless accounts for approximately 900 additional clicks. Second, display as a class achieved a higher CTR than video (*χ*²_1_=102.6; *P*<.001). Third, audio CTR exceeded display CTR even under the small-N caveat (*χ*²_1_=11.1; *P*=.001) but rests on only 5 observed audio clicks; the result is statistically present but fragile, and we treat it as hypothesis-generating rather than evidence of durable audio superiority. Within the display arm, mobile placements achieved approximately 3-fold higher CTR than desktop placements within each language (Table 2); the device-stratified impression split for display is not available in the per-creative export, so a parallel inferential test for device is not reported here.

### Audience Geography and Phase 2 Landing-Page Distribution

Impressions accumulated in expected Texas metropolitan areas with substantial Vietnamese American populations. The 5 largest cities accounted for approximately 60% (2,432,122/~4,019,000) of total impressions; together with the next 12 Texas cities they accounted for over 80% (3,210,788/~4,019,000; Table 4; full city-level distribution given in Table S7 in Multimedia Appendix 1). Approximately 4.7% (188,337/~4,019,000) of impressions were attributed to an “unknown” city. DV360 reports did not provide age or gender breakdowns at the user level; we therefore do not report age or gender stability across phases.

**Table 4.** Top cities by total impressions (all formats combined; February 15 to June 30, 2022).

City	Impressions, n	% of total
Houston	978,872	24.4
Dallas	799,977	19.9
Austin	316,715	7.9
San Antonio	185,974	4.6
Fort Worth	150,584	3.7
Other Texas cities (12+)	~1,283,000	~32.0
Unknown	188,337	4.7

During Phase 2 (May 26-June 30, 2022), GA Behavior Flow reporting captured 717 sessions with identifiable landing-page URLs. Of the 612 sessions that landed on a language-distinguished campaign page (excluding 105 sessions that landed on the homepage or an unlabeled page), 338 (55.2%) landed on a Vietnamese-language page, and 274 (44.8%) landed on an English-language page. The deviation from a 50:50 split favored Vietnamese-language pages (*χ*²_1_=6.7; *P*=.01); the Vietnamese vaccine-information landing page received the largest single-page share (n=225; 31.4% of Phase 2 sessions). Landing-page language is distinct from user browser language: our Table 5 reports browser language (21.9% Vietnamese in Phase 2), capturing a different signal, while the Behavior Flow data indicate that Phase 2 advertisement-driven traffic was directed to Vietnamese-language content for the majority of clickers regardless of their browser-side language settings (Table S10 in Multimedia Appendix 1).

**Table 5.** Website engagement metrics by campaign phase (February-June 2022)^a^.

Metric	Predisplay	Display phase	Before audio and video	Audio and video phase
Total visitors (N)	270	6437	133	780
Fold change from baseline	—^b^	23.84	—	5.87
Language distribution				
English-speaking users (%)	89.02	93.48	87.72	73.91
Vietnamese-speaking users (%)	9.59	6.07	8.77	21.85
Engagement quality				
Pages per session	2.61	1.74	1.53	2.2
Average session duration	104	10	9	15
Bounce rate (%)	67.89	45.87	57.5	10.46
Exit rate (%)	38.1	57.6	65.22	45.49
Page performance				
Total page views	496	11,528	184	1576
Average time on page (s)	1	14	18	13

a Language distribution reflects Google Analytics browser-language detection. The Phase 2 bounce rate of 10.46% is unusually low and may reflect measurement-window or small-N artefacts; we caution against direct comparison with the other bounce-rate cells.

b Not applicable.

Of all 717 Phase 2 Behavior Flow sessions, 673 (673/717, 93.9%) ended at the landing page without progressing to a second page; 44 sessions (44/717, 6.1%) progressed to a first interaction, and 18 (18/717, 2.5%) reached a second interaction. The high single-page rate is comparable to industry rates for digital-display-driven traffic and reflects the brevity of typical advertisement-click sessions.

### Website Engagement Analysis

The campaign drove traffic to PIVOT’s VietCOVID website during both campaign phases. During the static display campaign, website visitors increased from 270 (predisplay baseline) to 6,437, representing a 23.84-fold increase in traffic (Table 5). The audiovisual campaign period saw an increase from 133 to 780 visitors.

Language preference of website sessions, as captured by user browser settings, differed between phases. During the display phase, English-detected sessions comprised 93.48% of traffic and Vietnamese-detected sessions 6.07%. The audiovisual phase showed a shift, with English-detected sessions at 73.91% and Vietnamese-detected sessions rising to 21.85%. As noted under Audience Geography and Phase 2 Landing-Page Distribution, advertisement-driven landing-page language in Phase 2 was majority-Vietnamese even though browser-detected language remained English.

## Discussion

### Principal Results

Researchers partnered with CBOs to implement a bilingual digital health campaign using display, audio, and video advertisements for Vietnamese Americans in Texas about COVID-19 immunizations and participation in clinical trials, delivered through DV360 between February and June 2022, with a total budget of US $9472.54. The campaign delivered approximately 5 million impressions to roughly 1.4 million unique users in Phase 1 and 105,000 unique users in Phase 2, and was associated with substantial increases in traffic to an informational website during both phases.

Engaging community partners is essential in building trust and ensuring effectiveness as part of a public health campaign [31]. In following the continuum of community engagement in research, the researchers approached the CBOs to participate [32]. While researchers initiated the agenda, led the funding decisions, and informed messaging, the CBO created, ran, and tracked the campaign. Collaborating with CBOs who are trusted in the community and had technical and cultural expertise made this campaign more successful.

Additionally, high-quality, nuanced translation is an essential component of a successful campaign to reach the Vietnamese American population. The initial waves of immigrants left after the fall of Saigon in 1975 to escape Communism. The Vietnamese spoken in diaspora communities often reflects the state of that language used to communicate before they left Vietnam [33]. The Vietnamese American community, particularly older generations, holds deep anticommunist views to the point that accusations of communism can impact elections [34-36]. Thus, PIVOT translators were careful to avoid any terminology associated with post-1975 Vietnamese that could be construed as communist language and raise distrust. For example, “quý vị” was used in the campaign as a respectful term to address the audience. In the current Socialist Republic of Vietnam, “quí vị " would be used instead.

Moreover, the PIVOT graphic designer intentionally used images featuring people who appeared to be of different ages, genders, and East Asian or Southeast Asian descent. Representations of diversity can change the attention paid to an advertisement, influence recall, and affect attitudes and behavioral intentions [37]. The CBO had challenges finding freely available health education images featuring diverse Asian people, which aligns with previous findings about the need for stock photo libraries to improve their representation of historically marginalized populations [38].

Furthermore, using the DV360 platform was a cost-effective way to reach many people because it provides access to over 100 additional advertising exchanges through desktops, mobile devices, connected television, and digital radio. The frequency cap avoided overwhelming users with advertisements in which they are not interested. DV360 also offers dynamic creative optimization, which allows advertisements to change in real time based on the user’s behavior and preferences.

### Interpretation and Comparison With Prior Work

There is a long history of community engagement in health care and public health, supported by government agencies and other organizations, to address health disparities. CEAL was initiated to address challenges experienced by communities disproportionately affected by COVID-19 through community-engaged research, building trust, and promoting inclusive participation in National Institutes of Health (NIH)–led scientific responses [39]. This can serve as a model for working with communities on other health disparities.

Another benefit of the CEAL initiative is that partnering with the CBOs could strengthen internal structures, processes, decision-making systems, and resources to function. BPSOSH paid staff to participate in research through Collaborative Institutional Training Initiative (CITI Program), recruitment and consent of participants, and facilitating focus groups. PIVOT paid for a project manager, graphic designer, translator, and digital marketing consultant to develop the campaign. Investing in communities can build capacity for long-term engagement, trustworthiness, and addressing future challenges [40].

Public health learned lessons about how important science communication is during the COVID-19 pandemic. Providing science-based information in people’s preferred language is consequential to prebunk or address misinformation. Our CBO PIVOT’s Viet Fact Check project tackling misinformation started with COVID-19 and has been recognized for providing context and debunking online narratives in non-English languages [4,41].

Other CEAL teams also explored community-engaged digital outreach to educate different communities about COVID-19 immunizations. In Colorado, the CEAL team described working with Latino, Black/African American, rural African immigrant, and Urban American Indian/Alaska Native communities to transcreate COVID-19 vaccination campaigns with attention to messages, language, and imagery [42]. Similarly to their work, this project also centered messages on collective responsibility, featured multigenerational families, and avoided negative imagery.

Other CEAL teams also used videos to promote COVID-19 vaccination and clinical trial participation. A Puerto Rico CEAL group created and posted 34 short educational videos on their webpage to promote COVID-19 vaccination and reached nearly 3000 individuals through social media shares [43]. Louisiana CEAL worked with a Black-owned media firm to develop a multimedia campaign featuring a trusted local physician to promote clinical trial participation, which led to a 16-fold increase in Louisiana-CEAL website traffic and helped achieve more than 50% of a target trial enrollment goal [44]. Similarly, this project’s short educational videos featured health care professionals from the Vietnamese community.

California CEAL ran 2158 advertisements on digital, radio, and television that reached 29,013 people; 819 social media posts that received 3,317,904 reactions or comments; and developed 15 websites with 38,083 views [45]. This is reported as part of a broader paper summarizing activities by the entire California CEAL network, and it does not include the platforms used to serve the advertisements or posts. Ultimately, the various CEAL projects demonstrate cocreating campaigns to promote COVID-19 vaccines and participation in therapeutic trials with CBOs most likely made these more successful in raising awareness and engagement.

The most durable engagement signal in our data is the Vietnamese and mobile device usage in the display arm. Consistent with the Elaboration Likelihood Model, in-language messages delivered through the mobile devices that diaspora populations preferentially use for in-language media consumption produced higher click-through than central-route channels [29]. The pattern is also consistent with a previous observation that integrated, format-diverse digital campaigns for cultural minorities benefit from in-language and platform-appropriate placement rather than format substitution alone [46]. Successful cultural adaptation in digital health extends beyond translation to alignment with users’ platform and infrastructure realities [24]. Mobile-led digital adoption among racial minority populations during the pandemic appears to have somewhat narrowed preexisting digital divides—patient portal use among underprivileged racial minority groups rose 16%‐23% in one 25,612-patient longitudinal study, driven primarily by mobile adoption [47]; our findings on Vietnamese mobile display CTR are consistent with this broader pattern of mobile-substitution for desktop access in historically marginalized populations.

Paid digital advertising has been associated with increased first-dose COVID-19 vaccinations at the population level in the US Department of Health and Human Services (HHS) “We Can Do This” campaign evaluation [48]. Recent field-experimental work in a 947,818-user Facebook trial demonstrated that simple structural choices in advertisement framing, such as question-and-answer versus direct statements, produce 9%‐11% improvements in clicks-per-dollar for vaccine messaging, suggesting that our cost metrics could be further optimized through structural framing improvements at no additional media budget [49]. The campaign’s 23.8-fold display-phase increase in website traffic indicates substantial short-term campaign-associated traffic growth. Previous research provides a useful point of reference for equity-oriented multilingual public-health messaging on social media engagement and recruitment outcomes [16-18]. Direct comparison with prior studies is limited by differences in platform, baseline traffic, target population, and outcome definitions.

Some public health campaigns have used Google Ads and measured engagement. Australian researchers used Google Ads to promote a suicide crisis hotline that resulted in a CTR of 4.02% and an average cost of US $13.57 per helpline call [50]. Researchers in Oregon used Google Ads to measure a lethal means safety campaign that ran 275 days and resulted in 1,849,077 impressions and a CTR of 18.5% [51]. These may have had higher CTR due to the targeted advertisement display of users searching suicide-related terms.

Other studies have used online advertising to recruit for clinical trials. Google and Facebook advertisements were used to recruit participants for a clinical study monitoring COVID-19 using wearable devices and found traditional recruitment through email, electronic direct message, paper advertisements, and word-of-mouth was more successful [52]. Ibis Reproductive Health compared Facebook, Google Ads, and Reddit (Reddit, Inc) as recruitment platforms for a study on abortion-seeking experience in the United States and found Facebook more time- and cost-effective [53]. Further experimentation is needed to determine how to effectively use these accessible platforms to reach specific populations and promote desired behaviors.

While there has been a significant focus on public health campaigns using social media advertisements, few studies in the literature describe using the DV360 platform [54,55]. American researchers and an Indian nonprofit institution used DV360 to run advertisements to promote a tobacco cessation app and found a CTR of 5% with 5111 app downloads [56]. With Google’s extensive network, campaigns can place advertisements across many websites and platforms to benefit from real-time, automated bidding for cost-effective advertisement placement.

Since this campaign ran, the emergence of new technologies like AI could make reaching different populations easier. Most research on AI in public health campaigns has been focused more on audience segmentation and impact assessment rather than campaign design [57]. Some researchers have described ways AI could be used to support public health campaigns: design campaign proposals, create natural-sounding messages, predictive analytics to identify high-risk individuals, personalized interventions tailored to specific needs of the individual, social network analysis to map connections and influences within communities, and provide real-time monitoring and feedback to individuals [58-60].

Additionally, since this campaign started, more translation tools using large language models (LLMs) have become available for general usage. Concerns around the accuracy of translation and navigating cultural values are considerations in building trust with users [61,62]. LLM and machine translation (MT) are improving, but human translation is more complex and accepted when Spanish, Chinese, and Hungarian have been examined [63-65]. Other ethical issues arising from using these technologies include undermining the value of human translators and developing and training MT systems [66]. Including translators from the desired community in the development of translation tools can help identify priorities based on needs and specificity and evaluate the tools’ performance [67].

However, it must be noted that AI carries the risk of rapidly spreading misinformation undermining trust in health systems and causing widespread nonadherence to critical public health measures like immunizations [68]. AI tools can perpetuate systemic biases related to gender, race and ethnicity, facial expressions, and appearances [69]. There are also concerns about the water and energy consumption by data centers supporting AI use and their impact on the environment [70,71]. Care and caution must be taken in using AI tools.

### Limitations

Several limitations warrant consideration. First, DV360 and GA do not verify user ethnicity, age, or gender; reach to Vietnamese American users is inferred from language-and-locale signals, contextual placement, and Phase 2 audience-segment targeting. Because click-through is measured on consistent advertisement content, ethnic misclassification of English-arm clickers should attenuate rather than inflate any Vietnamese American–specific effect, biasing English-arm estimates toward the null. The audience-stability claim is therefore restricted to geographic distribution and language settings.

Second, sequential phasing and opaque automated placement confound format comparisons. Display ran in February-March and audiovisual in May-June, and DV360’s bidder selected specific apps and time slots without per-impression transparency. The design supports comparative description within each phase but not causal attribution to format alone; the top-app analysis (Tables S5 and S6 in Multimedia Appendix 1) is the strongest available placement transparency.

Third, the audio impression base is small (≈1800 total) and audio CTRs have correspondingly wide CIs; the May 2022 peak should not be overinterpreted.

Fourth, we did not measure downstream vaccination uptake or clinical trial enrollment; the outcomes reported are exposure and engagement metrics only, and our findings should not be read as evidence of behavior change [23,24,72]. No postengagement behavioral data linked clicks to vaccination or trial enrollment. Future campaigns could bridge this gap via campaign-unique landing URLs with opt-in handoff to a partner clinic’s electronic health record (EHR) system, brief on-page intake of vaccination status or intent, or zip-code-level linkage to public-health vaccination dashboards. Our group’s parallel cohort work shows that digital-only engagement strategies underrepresent primary Vietnamese speakers, female respondents, and individuals without a college degree [72]; platform-side metrics therefore likely understate reach to the most vulnerable subpopulations.

Fifth, the revised advertisements were not pilot-tested before the February 15 launch; the focus-group review with BPSOSH was the only prelaunch validation.

Sixth, digital advertising platforms remain vulnerable to the broader infodemic [1]. The Vietnamese American diaspora’s use of encrypted apps such as Zalo developed by VNG Corporation for intergenerational health-information sharing is largely opaque to platform-level outreach; targeted disinformation—including coordinated narratives circulated through diaspora-specific networks—further undermines platform-only approaches [73]. Complementary grassroots and trusted-messenger strategies, including infodemiology-informed dynamic content adaptation, remain necessary [73-75].

### Conclusions

Digital health campaigns have become increasingly necessary for reaching diverse populations, particularly during public health emergencies. This study describes the feasibility, engagement patterns, and platform-level placement characteristics of an integrated digital marketing platform delivering science-based public health communications to Vietnamese Americans in Texas.

### Practical Recommendations for Implementation

Public health practitioners developing digital campaigns to engage Vietnamese American communities could consider 3 key strategies. First, plan for in-language creative to outperform English creative within static display, and budget for in-language content as a primary channel rather than as a translation afterthought. Second, plan for mobile to outperform desktop within each language, and design creative for vertical orientation, short captions, and mobile-native interaction rather than treating mobile as a desktop variant. Third, plan for language-of-engagement to vary across formats and campaign stages rather than presupposing a single language ratio, and monitor in-campaign engagement to reallocate spend accordingly.

Audio placements achieved high click-through in 1 month of our campaign but on a small impression base that did not replicate, and we do not recommend prioritizing audio over in-language mobile display based on current data. Replication with audio impression budgets 30 to 60 times larger would be required to assess audio’s durable value for this population. Campaign teams with limited resources should prioritize in-language mobile display. Our cost metrics—average CPC US $2.35 and CPM US $1.45—are favorable relative to general population health-outreach benchmarks and may serve as a planning reference for similar campaigns.

### Future Directions and Next Steps

Three concrete extensions follow. First, future campaigns should precommit to an engagement-to-behavior linkage at the design stage—through campaign-unique landing URLs with opt-in handoff to partner clinics, on-page self-reporting of vaccination status or intent, or zip-code-level linkage to public health data [72]. Second, larger audio impression budgets (estimated at 30‐60× the current allocation) are needed before audio’s role for diaspora populations can be evaluated against in-language mobile display. Third, infodemiology-informed dynamic content adjustment—adapting creative and placement in response to real-time misinformation signals—represents a methodologically tractable extension of the current design [73-75]. Replicating recent structural-framing interventions (such as question and answer [Q&A] vs direct statements) in culturally tailored, multilingual contexts would be a tractable, cost-effective extension [49]. Health care organizations and public health agencies can immediately apply these findings by incorporating in-language, mobile-first design into their digital strategies, investing in robust analytics to track engagement across languages and platforms, and precommitting to mechanisms that link digital engagement to downstream health outcomes.

## Supplementary material

10.2196/90301 Multimedia Appendix 1 Additional information referenced about campaign development, app placement, impressions, and analytics.

10.2196/90301 Multimedia Appendix 2 English language video advertisement about COVID-19 vaccination.

10.2196/90301 Multimedia Appendix 3 Vietnamese language video advertisement about COVID-19 vaccination.

10.2196/90301 Multimedia Appendix 4 Vietnamese language video advertisement about participating in COVID-19 clinical trials.

10.2196/90301 Multimedia Appendix 5 English language video advertisement about participating in COVID-19 clinical trials. 1

10.2196/90301 Checklist 1 CONSORT-EHEALTH checklist.

## Abbreviations

BPSOSH: Boat People SOS-Houston
CARE: Stanford Center for Asian Health Research and Education
CBO: community-based organization
CEAL: Community-Engagement Research Alliance
CITI: Collaborative Institutional Training Initiative
CONSORT-EHEALTH: Consolidated Standards of Reporting Trials of Electronic and Mobile Health Applications and Online Telehealth
CPC: cost-per-click
CPM: cost-per-thousand impressions
CPV: cost-per-view
CTR: click-through rate
DV360: Google’s Display & Video 360 advertising platform
EHR: electronic health record
ELM: Elaboration Likelihood Model
GA: Google Analytics
HBM: Health Belief Model
HEAL: Helping to End Addiction Long-Term
HHS: US Department of Health and Human Services
LLM: large language model
MT: machine translation
NIH: National Institutes of Health
PMT: Protection Motivation Theory
Q&A: question and answer
