# Supplementary material for: Digital Campaign to Promote COVID-19 Vaccination and Clinical Trial Participation Among Vietnamese Americans: Observational Engagement Study
Source: J Med Internet Res. 2026 Oct 15;28:e90301. doi: 10.2196/90301 (PMC13689936; doi:10.2196/90301)
Supplement: Supplementary file 1 [file jmir-v28-e90301-s006.pdf]

CONSORT-EHEALTH (V 1.6.1) - Submission/Publication Form

The CONSORT-EHEALTH checklist is intended for authors of randomized trials evaluating web-based and Internet-based applications/interventions, including mobile interventions, electronic games (incl multiplayer games), social media, certain telehealth applications, and other interactive and/or networked electronic applications. Some of the items (e.g. all subitems under item 5 - description of the intervention) may also be applicable for other study designs.

The goal of the CONSORT EHEALTH checklist and guideline is to be
a) a guide for reporting for authors of RCTs,
b) to form a basis for appraisal of an ehealth trial (in terms of validity)

CONSORT-EHEALTH items/subitems are MANDATORY reporting items for studies published in the Journal of Medical Internet Research and other journals / scientific societies endorsing the checklist.

Items numbered 1., 2., 3., 4a., 4b etc are original CONSORT or CONSORT-NPT (non-pharmacologic treatment) items.
Items with Roman numerals (i., ii, iii, iv etc.) are CONSORT-EHEALTH extensions/clarifications.

As the CONSORT-EHEALTH checklist is still considered in a formative stage, we would ask that you also RATE ON A SCALE OF 1-5 how important/useful you feel each item is FOR THE PURPOSE OF THE CHECKLIST and reporting guideline (optional).

Mandatory reporting items are marked with a red *.
In the textboxes, either copy & paste the relevant sections from your manuscript into this form - please include any quotes from your manuscript in QUOTATION MARKS,
or answer directly by providing additional information not in the manuscript, or elaborating on why the item was not relevant for this study.

YOUR ANSWERS WILL BE PUBLISHED AS A SUPPLEMENTARY FILE TO YOUR PUBLICATION IN JMIR AND ARE CONSIDERED PART OF YOUR PUBLICATION (IF ACCEPTED).
Please fill in these questions diligently. Information will not be copyedited, so please use proper spelling and grammar, use correct capitalization, and avoid abbreviations.

DO NOT FORGET TO SAVE AS PDF _AND_ CLICK THE SUBMIT BUTTON SO YOUR ANSWERS ARE IN OUR DATABASE !!!

Citation Suggestion (if you append the pdf as Appendix we suggest to cite this paper in the caption):

Eysenbach G, CONSORT-EHEALTH Group
CONSORT-EHEALTH: Improving and Standardizing Evaluation Reports of Web-based and Mobile Health Interventions
J Med Internet Res 2011;13(4):e126
URL: http://www.jmir.org/2011/4/e126/
doi: 10.2196/jmir.1923
PMID: 22209829

Sign in to Google to save your progress. Learn more

* Indicates required question

Your name *
First Last

Bich-May Nguyen

Primary Affiliation (short), City, Country *
University of Toronto, Toronto, Canada

University of Houston, Houston, United States

Your e-mail address *
abc@gmail.com

bnguyen49@uh.edu

Title of your manuscript *

Provide the (draft) title of your manuscript.

Digital Campaign to Promote COVID-19 Vaccination and Clinical Trial Participation Among Vietnamese Americans: An Observational Engagement Study

Name of your App/Software/Intervention *

If there is a short and a long/alternate name, write the short name first and add the long name in brackets.

digital outreach campaign using Google's Disp

Evaluated Version (if any)

e.g. "V1", "Release 2017-03-01", "Version 2.0.27913"

Revision 2

Language(s) *

What language is the intervention/app in? If multiple languages are available, separate by comma (e.g. "English, French")

English, Vietnamese

URL of your Intervention Website or App

e.g. a direct link to the mobile app on app in appstore (itunes, Google Play), or URL of the website. If the intervention is a DVD or hardware, you can also link to an Amazon page.

Your answer

URL of an image/screenshot (optional)

Your answer

Accessibility *

Can an enduser access the intervention presently?

- ○ access is free and open
- ○ access only for special usergroups, not open
- ○ access is open to everyone, but requires payment/subscription/in-app purchases
- ◉ app/intervention no longer accessible
- ○ Other:

Primary Medical Indication/Disease/Condition *

e.g. "Stress", "Diabetes", or define the target group in brackets after the condition, e.g. "Autism (Parents of children with)", "Alzheimers (Informal Caregivers of)"

COVID-19

Primary Outcomes measured in trial *

comma-separated list of primary outcomes reported in the trial

impressions, unique users, cost per click, cost

Secondary/other outcomes

Are there any other outcomes the intervention is expected to affect?

Your answer

Recommended "Dose" *

What do the instructions for users say on how often the app should be used?

- ○ Approximately Daily
- ○ Approximately Weekly
- ○ Approximately Monthly
- ○ Approximately Yearly
- ○ "as needed"
- ◉ Other: ads served 12 times to unique user during duration of campaign

Approx. Percentage of Users (starters) still using the app as recommended after 3 months *

- ○ unknown / not evaluated
- ◉ 0-10%
- ○ 11-20%
- ○ 21-30%
- ○ 31-40%
- ○ 41-50%
- ○ 51-60%
- ○ 61-70%
- ○ 71%-80%
- ○ 81-90%
- ○ 91-100%
- ○ Other:

Overall, was the app/intervention effective? *

- ○ yes: all primary outcomes were significantly better in intervention group vs control
- ○ partly: SOME primary outcomes were significantly better in intervention group vs control
- ○ no statistically significant difference between control and intervention
- ○ potentially harmful: control was significantly better than intervention in one or more outcomes
- ◉ inconclusive: more research is needed
- ○ Other:

Article Preparation Status/Stage *

At which stage in your article preparation are you currently (at the time you fill in this form)

- ○ not submitted yet - in early draft status
- ○ not submitted yet - in late draft status, just before submission
- ○ submitted to a journal but not reviewed yet
- ○ submitted to a journal and after receiving initial reviewer comments
- ○ submitted to a journal and accepted, but not published yet
- ○ published
- ◉ Other: submitted to a journal and after receiving second round of reviewer cc

Journal *

If you already know where you will submit this paper (or if it is already submitted), please provide the journal name (if it is not JMIR, provide the journal name under "other")

- ○ not submitted yet / unclear where I will submit this
- ◉ Journal of Medical Internet Research (JMIR)
- ○ JMIR mHealth and UHealth
- ○ JMIR Serious Games
- ○ JMIR Mental Health
- ○ JMIR Public Health
- ○ JMIR Formative Research
- ○ Other JMIR sister journal
- ○ Other:

Is this a full powered effectiveness trial or a pilot/feasibility trial? *

- ◉ Pilot/feasibility
- ○ Fully powered

Manuscript tracking number *

If this is a JMIR submission, please provide the manuscript tracking number under "other" (The ms tracking number can be found in the submission acknowledgement email, or when you login as author in JMIR. If the paper is already published in JMIR, then the ms tracking number is the four-digit number at the end of the DOI, to be found at the bottom of each published article in JMIR)

- ○ no ms number (yet) / not (yet) submitted to / published in JMIR
- ◉ Other: #90301

TITLE AND ABSTRACT

1a) TITLE: Identification as a randomized trial in the title

1a) Does your paper address CONSORT item 1a? *

I.e does the title contain the phrase "Randomized Controlled Trial"? (if not, explain the reason under "other")

- ○ yes
- ◉ Other: No, it is not a randomized controlled trial. "An Observational Engagem

1a-i) Identify the mode of delivery in the title

Identify the mode of delivery. Preferably use “web-based” and/or “mobile” and/or “electronic game” in the title. Avoid ambiguous terms like “online”, “virtual”, “interactive”. Use “Internet-based” only if Intervention includes non-web-based Internet components (e.g. email), use “computer-based” or “electronic” only if offline products are used. Use “virtual” only in the context of “virtual reality” (3-D worlds). Use “online” only in the context of “online support groups”. Complement or substitute product names with broader terms for the class of products (such as “mobile” or “smart phone” instead of “iphone”), especially if the application runs on different platforms.

	1	2	3	4	5	
subitem not at all important	○	○	○	○	○	essential

Does your paper address subitem 1a-i? *

Copy and paste relevant sections from manuscript title (include quotes in quotation marks "like this" to indicate direct quotes from your manuscript), or elaborate on this item by providing additional information not in the ms, or briefly explain why the item is not applicable/relevant for your study

"Digital Campaign" used since the Google's Display & Video 360 served ads across internet sites, desktops, electronic devices, mobile apps, connected television, and digital radio.

1a-ii) Non-web-based components or important co-interventions in title

Mention non-web-based components or important co-interventions in title, if any (e.g., “with telephone support”).

	1	2	3	4	5	
subitem not at all important	○	○	○	○	○	essential

Does your paper address subitem 1a-ii?

Copy and paste relevant sections from manuscript title (include quotes in quotation marks "like this" to indicate direct quotes from your manuscript), or elaborate on this item by providing additional information not in the ms, or briefly explain why the item is not applicable/relevant for your study

Your answer

1a-iii) Primary condition or target group in the title

Mention primary condition or target group in the title, if any (e.g., “for children with Type I Diabetes”) Example: A Web-based and Mobile Intervention with Telephone Support for Children with Type I Diabetes: Randomized Controlled Trial

	1	2	3	4	5	
subitem not at all important	○	○	○	○	○	essential

Does your paper address subitem 1a-iii? *

Copy and paste relevant sections from manuscript title (include quotes in quotation marks "like this" to indicate direct quotes from your manuscript), or elaborate on this item by providing additional information not in the ms, or briefly explain why the item is not applicable/relevant for your study

Yes, "COVID-19 Vaccination and Clinical Trial Participation Among Vietnamese Americans"

1b) ABSTRACT: Structured summary of trial design, methods, results, and conclusions

NPT extension: Description of experimental treatment, comparator, care providers, centers, and blinding status.

1b-i) Key features/functionalities/components of the intervention and comparator in the METHODS section of the ABSTRACT

Mention key features/functionalities/components of the intervention and comparator in the abstract. If possible, also mention theories and principles used for designing the site. Keep in mind the needs of systematic reviewers and indexers by including important synonyms. (Note: Only report in the abstract what the main paper is reporting. If this information is missing from the main body of text, consider adding it)

	1	2	3	4	5	
subitem not at all important	○	○	○	○	○	essential

Does your paper address subitem 1b-i? *

Copy and paste relevant sections from the manuscript abstract (include quotes in quotation marks "like this" to indicate direct quotes from your manuscript), or elaborate on this item by providing additional information not in the ms, or briefly explain why the item is not applicable/relevant for your study

"We implemented a two-phase digital campaign on Google's Display & Video 360: static display ads (Phase 1: February 15 – March 31, 2022) and audiovisual content (Phase 2: May 26 – June 30, 2022). With community-based organizations, six static displays, two videos, and two audio ads were developed in English and Vietnamese using focus group review and back-translation. Ad placements were constrained to a Texas Vietnamese audience segment. Total budget was $9,472.54."

1b-ii) Level of human involvement in the METHODS section of the ABSTRACT

Clarify the level of human involvement in the abstract, e.g., use phrases like “fully automated” vs. “therapist/nurse/care provider/physician-assisted” (mention number and expertise of providers involved, if any). (Note: Only report in the abstract what the main paper is reporting. If this information is missing from the main body of text, consider adding it)

	1	2	3	4	5	
subitem not at all important	○	○	○	○	○	essential

Does your paper address subitem 1b-ii?

Copy and paste relevant sections from the manuscript abstract (include quotes in quotation marks "like this" to indicate direct quotes from your manuscript), or elaborate on this item by providing additional information not in the ms, or briefly explain why the item is not applicable/relevant for your study

Your answer

1b-iii) Open vs. closed, web-based (self-assessment) vs. face-to-face assessments in the METHODS section of the ABSTRACT

Mention how participants were recruited (online vs. offline), e.g., from an open access website or from a clinic or a closed online user group (closed usergroup trial), and clarify if this was a purely web-based trial, or there were face-to-face components (as part of the intervention or for assessment). Clearly say if outcomes were self-assessed through questionnaires (as common in web-based trials). Note: In traditional offline trials, an open trial (open-label trial) is a type of clinical trial in which both the researchers and participants know which treatment is being administered. To avoid confusion, use “blinded” or “unblinded” to indicated the level of blinding instead of “open”, as “open” in web-based trials usually refers to “open access” (i.e. participants can self-enrol). (Note: Only report in the abstract what the main paper is reporting. If this information is missing from the main body of text, consider adding it)

	1	2	3	4	5	
subitem not at all important	○	○	○	○	○	essential

Does your paper address subitem 1b-iii?

Copy and paste relevant sections from the manuscript abstract (include quotes in quotation marks "like this" to indicate direct quotes from your manuscript), or elaborate on this item by providing additional information not in the ms, or briefly explain why the item is not applicable/relevant for your study

Your answer

1b-iv) RESULTS section in abstract must contain use data

Report number of participants enrolled/assessed in each group, the use/uptake of the intervention (e.g., attrition/adherence metrics, use over time, number of logins etc.), in addition to primary/secondary outcomes. (Note: Only report in the abstract what the main paper is reporting. If this information is missing from the main body of text, consider adding it)

	1	2	3	4	5	
subitem not at all important	○	○	○	○	○	essential

Does your paper address subitem 1b-iv?

Copy and paste relevant sections from the manuscript abstract (include quotes in quotation marks "like this" to indicate direct quotes from your manuscript), or elaborate on this item by providing additional information not in the ms, or briefly explain why the item is not applicable/relevant for your study

Your answer

1b-v) CONCLUSIONS/DISCUSSION in abstract for negative trials

Conclusions/Discussions in abstract for negative trials: Discuss the primary outcome - if the trial is negative (primary outcome not changed), and the intervention was not used, discuss whether negative results are attributable to lack of uptake and discuss reasons. (Note: Only report in the abstract what the main paper is reporting. If this information is missing from the main body of text, consider adding it)

	1	2	3	4	5	
subitem not at all important	○	○	○	○	○	essential

Does your paper address subitem 1b-v?

Copy and paste relevant sections from the manuscript abstract (include quotes in quotation marks "like this" to indicate direct quotes from your manuscript), or elaborate on this item by providing additional information not in the ms, or briefly explain why the item is not applicable/relevant for your study

Your answer

INTRODUCTION

2a) In INTRODUCTION: Scientific background and explanation of rationale

2a-i) Problem and the type of system/solution

Describe the problem and the type of system/solution that is object of the study: intended as stand-alone intervention vs. incorporated in broader health care program? Intended for a particular patient population? Goals of the intervention, e.g., being more cost-effective to other interventions, replace or complement other solutions? (Note: Details about the intervention are provided in “Methods” under 5)

	1	2	3	4	5	
subitem not at all important	○	○	○	○	○	essential

Does your paper address subitem 2a-i? *

Copy and paste relevant sections from the manuscript (include quotes in quotation marks "like this" to indicate direct quotes from your manuscript), or elaborate on this item by providing additional information not in the ms, or briefly explain why the item is not applicable/relevant for your study

"The COVID-19 pandemic precipitated a global health crisis and an unprecedented "infodemic" - a surge of information, including false or misleading content, that complicated public health communication efforts [1]. This challenge was particularly acute for non-English speaking communities in the United States, who faced unique barriers in accessing reliable health information. Asian Americans, specifically Vietnamese Americans, encountered additional obstacles due to language barriers, cultural differences, and heightened discrimination during the pandemic [2]. The Asian American population, of which Vietnamese Americans are one of the largest origin groups, comprises approximately 25 million people and is characterized by high mobile-device adoption and significant within-group heterogeneity in language preference and digital access [3]."

2a-ii) Scientific background, rationale: What is known about the (type of) system

Scientific background, rationale: What is known about the (type of) system that is the object of the study (be sure to discuss the use of similar systems for other conditions/ diagnoses, if appropiate), motivation for the study, i.e. what are the reasons for and what is the context for this specific study, from which stakeholder viewpoint is the study performed, potential impact of findings [2]. Briefly justify the choice of the comparator.

	1	2	3	4	5	
subitem not at all important	○	○	○	○	○	essential

Does your paper address subitem 2a-ii? *

Copy and paste relevant sections from the manuscript (include quotes in quotation marks "like this" to indicate direct quotes from your manuscript), or elaborate on this item by providing additional information not in the ms, or briefly explain why the item is not applicable/relevant for your study

"Recent studies have highlighted how the rapid spread of misinformation through social media and online platforms disproportionately affected Asian American communities. Chong et al. documented how COVID-19 misinformation circulated through subgroup-specific, in-language social media platforms — WeChat among Chinese Americans, KakaoTalk and YouTube among Korean Americans, and Facebook and YouTube among Vietnamese Americans — and linked this exposure to potential downstream effects on vaccine decision-making and health protective behaviors [4]. These challenges were compounded for Vietnamese Americans, who report lower rates of English proficiency compared to other Asian American groups [5].

Digital health technologies emerged as crucial tools for disseminating public health information during the pandemic. However, their effectiveness varied significantly based on cultural and linguistic adaptation. A 2023 systematic review of 34 studies on digital health among culturally and linguistically diverse populations found that platforms theoretically capable of democratizing access often fail in implementation due to gaps in digital literacy, culturally inappropriate interface design, and language exclusion [6]. Specifically within Asian American communities, Quach et al. found that 56.1% of surveyed Asian American respondents had limited English proficiency, and the study linked those language access constraints to low COVID-19 testing uptake, underscoring the need for multilingual outreach strategies [7]. Community-based organizations (CBOs) have played a vital role in bridging these gaps, with Wong et al. describing how Asian American–serving CBOs delivered culturally and linguistically centered COVID-19 outreach — including in-language social media content, webinars, and community health worker programs — reaching tens of thousands of community members [8].

Digital marketing platforms offer opportunities to reach diverse populations, but the literature systematically evaluating their effectiveness for multilingual health campaigns across multiple formats remains limited. Peer-reviewed CTR benchmarks for multilingual public health campaigns remain limited and highly platform-dependent. Reviews of public health digital-campaign evaluation describe CTR as a commonly used proximal engagement metric while emphasizing that values vary widely across platforms and do not by themselves establish behavior change [10]. In the HEALing Communities Study, paid social-media health advertisements had higher CTR than banner/display advertisements, with banner/display median CTRs of approximately 0.08–0.13% across analyses[9]. Adjacent multilingual campaigns have reported higher CTRs in translated-video and search-ad contexts — translated Vietnamese/Mandarin Facebook video outreach yielded higher CTR than English-language posts (0.77% vs 0.62%)[11], and multilingual Google AdWords recruitment to a health research website found CTR varied substantially by language[12]. Recent work has examined digital outreach to broader racial and ethnic populations: Page-Tan and Hope analyzed COVID-19 testing and vaccine Twitter posts from Houston government agencies, elected officials, and CBOs, finding that government agencies were most likely to address access, fear, and language barriers [15]; Bonnevie et al. evaluated a social-media micro-influencer flu-vaccine campaign among African American and Hispanic audiences, reporting 117 influencers, 69,495 engagements, and improvements in flu-vaccine beliefs and community-attitude measures [16]. The Stanford Center for Asian Health Research and Education conducted a 10-week social media campaign across Facebook, Instagram, and Twitter that increased the percentage of Asian participants in the Stanford Research Registry from 14.3% to 23.8% (525 to 1,871 individuals), providing one of the few recent quantitative benchmarks for digital outreach to Asian American populations

specifically [17]. Park and colleagues have explored digital recruitment strategies for Asian Americans and Pacific Islanders more broadly [18]. However, few studies have systematically evaluated different digital formats — static, video, and audio — specifically in reaching Vietnamese American audiences during public health emergencies, and the format-versus-other-design-parameters question (language, device, placement) has received little empirical attention."

2b) In INTRODUCTION: Specific objectives or hypotheses

Does your paper address CONSORT subitem 2b? *

Copy and paste relevant sections from the manuscript (include quotes in quotation marks "like this" to indicate direct quotes from your manuscript), or elaborate on this item by providing additional information not in the ms, or briefly explain why the item is not applicable/relevant for your study

"This study describes a community-engaged digital campaign directed at Vietnamese Americans in Texas to promote COVID-19 vaccinations and clinical trial participation. Using an online marketing platform, we evaluate the engagement and placement characteristics of multiple digital advertising formats in English and Vietnamese, providing observational insights into design parameters that govern engagement for linguistically diverse diaspora populations."

METHODS

3a) Description of trial design (such as parallel, factorial) including allocation ratio

Does your paper address CONSORT subitem 3a? *

Copy and paste relevant sections from the manuscript (include quotes in quotation marks "like this" to indicate direct quotes from your manuscript), or elaborate on this item by providing additional information not in the ms, or briefly explain why the item is not applicable/relevant for your study

"This observational study evaluated a bilingual digital health campaign focused on Vietnamese Americans in Texas implemented between February 15 and June 30, 2022. The campaign was structured in two distinct phases: an initial display advertisement phase from February 15 to March 31, 2022, followed by an audiovisual content phase from May 26 to June 30, 2022. The total campaign budget was $9,472.54, with $5,879.42 allocated to display advertisements and $3,593.12 to YouTube, connected television, and digital-radio audio content. The Vietnamese American Texas cohort recruited as part of this campaign overlaps with the cohort described in our group's companion methodological paper on survey modalities and vaccine uptake [19]; the same community-based organization (CBO) partnerships — PIVOT and Boat People SOS-Houston (BPSOSH) — supported recruitment and community engagement for both efforts."

3b) Important changes to methods after trial commencement (such as eligibility criteria), with reasons

Does your paper address CONSORT subitem 3b? *

Copy and paste relevant sections from the manuscript (include quotes in quotation marks "like this" to indicate direct quotes from your manuscript), or elaborate on this item by providing additional information not in the ms, or briefly explain why the item is not applicable/relevant for your study

Not applicable. We did not make changes to methods after trial commencement.

3b-i) Bug fixes, Downtimes, Content Changes

Bug fixes, Downtimes, Content Changes: ehealth systems are often dynamic systems. A description of changes to methods therefore also includes important changes made on the intervention or comparator during the trial (e.g., major bug fixes or changes in the functionality or content) (5-iii) and other “unexpected events” that may have influenced study design such as staff changes, system failures/downtimes, etc. [2].

	1	2	3	4	5	
subitem not at all important	○	○	○	○	○	essential

Does your paper address subitem 3b-i?

Copy and paste relevant sections from the manuscript (include quotes in quotation marks "like this" to indicate direct quotes from your manuscript), or elaborate on this item by providing additional information not in the ms, or briefly explain why the item is not applicable/relevant for your study

Your answer

4a) Eligibility criteria for participants

Does your paper address CONSORT subitem 4a? *

Copy and paste relevant sections from the manuscript (include quotes in quotation marks "like this" to indicate direct quotes from your manuscript), or elaborate on this item by providing additional information not in the ms, or briefly explain why the item is not applicable/relevant for your study

"Explicit targeting parameters were:
• Geography: users located in the state of Texas.
• Language signals: device or session language preference set to English (for English creatives) or Vietnamese (for Vietnamese creatives).
• Audience segment (Phase 2 only): in the video/radio/YouTube phase (May 26 – June 30, 2022), placements were additionally constrained to a “Texas Vietnamese” audience segment of approximately 300,000 users defined by DV360’s language-and-locale signals.
• Contextual signals: PIVOT staff with prior experience in Vietnamese American digital outreach assembled a list of topic- and language-relevant contextual keywords used by DV360’s contextual-targeting layer to identify content pages and applications likely to be consumed by Vietnamese American users in Texas. Keywords spanned six categories: (1) Vietnamese-language news and media; (2) family- and parenting-related content; (3) education and learning content; (4) Vietnamese-language entertainment; (5) general COVID-19 and health-information categories; and (6) clinical-research and trial-related terms. The list was assembled prior to launch from prior community-engagement campaigns by the same CBO partner and was not retained in archived form by the research team. Keyword effectiveness was monitored indirectly through the platform’s placement reports; the top-app and top-website distributions for each language arm (Supplementary Tables S4 and S5) provide direct evidence of where contextual signals routed impressions. We note that contextual keywords were one of several inputs to placement; the DV360 bidder integrated geographic constraint, language signal, frequency cap, audience-segment overlay (Phase 2), and keyword signal algorithmically. We therefore describe the keyword strategy at the level of category and effect rather than enumerating individual terms.
• Frequency cap: twelve impressions per unique user across the campaign period.
• Creative rotation: DV360’s auto-optimization was enabled, with the platform giving heavier delivery to better-performing creatives."

4a-i) Computer / Internet literacy

Computer / Internet literacy is often an implicit “de facto” eligibility criterion - this should be explicitly clarified.

	1	2	3	4	5	
subitem not at all important	○	○	○	○	○	essential

Does your paper address subitem 4a-i?

Copy and paste relevant sections from the manuscript (include quotes in quotation marks "like this" to indicate direct quotes from your manuscript), or elaborate on this item by providing additional information not in the ms, or briefly explain why the item is not applicable/relevant for your study

Your answer

4a-ii) Open vs. closed, web-based vs. face-to-face assessments:

Open vs. closed, web-based vs. face-to-face assessments: Mention how participants were recruited (online vs. offline), e.g., from an open access website or from a clinic, and clarify if this was a purely web-based trial, or there were face-to-face components (as part of the intervention or for assessment), i.e., to what degree got the study team to know the participant. In online-only trials, clarify if participants were quasi-anonymous and whether having multiple identities was possible or whether technical or logistical measures (e.g., cookies, email confirmation, phone calls) were used to detect/prevent these.

	1	2	3	4	5	
subitem not at all important	○	○	○	○	○	essential

Does your paper address subitem 4a-ii? *

Copy and paste relevant sections from the manuscript (include quotes in quotation marks "like this" to indicate direct quotes from your manuscript), or elaborate on this item by providing additional information not in the ms, or briefly explain why the item is not applicable/relevant for your study

Not applicable. No recruitment was completed. We directed digital ads using explicit parameters based on geography, language signals, and audience segment for video and audio ads as described in 4a.

4a-iii) Information giving during recruitment

Information given during recruitment. Specify how participants were briefed for recruitment and in the informed consent procedures (e.g., publish the informed consent documentation as appendix, see also item X26), as this information may have an effect on user self-selection, user expectation and may also bias results.

	1	2	3	4	5	
subitem not at all important	◯	◯	◯	◯	◯	essential

Does your paper address subitem 4a-iii?

Copy and paste relevant sections from the manuscript (include quotes in quotation marks "like this" to indicate direct quotes from your manuscript), or elaborate on this item by providing additional information not in the ms, or briefly explain why the item is not applicable/relevant for your study

Your answer

4b) Settings and locations where the data were collected

Does your paper address CONSORT subitem 4b? *

Copy and paste relevant sections from the manuscript (include quotes in quotation marks "like this" to indicate direct quotes from your manuscript), or elaborate on this item by providing additional information not in the ms, or briefly explain why the item is not applicable/relevant for your study

"Performance data were collected through DV360 and Google Analytics (GA) platforms using standardized digital marketing metrics. Key engagement metrics included impressions (the number of times ads were displayed to a user), reach (the total number of viewers), clicks (user interactions with ads), and click-through rate (CTR, calculated as clicks divided by impressions). For video content delivered through YouTube, connected television, and YouTube-engine partners, impressions were counted using Google's TrueView standard, which records a view only when a viewer watches at least 80% of the advertisement. Digital radio advertisements were counted as impressions when the audio played for any duration. Financial performance was tracked through cost per click (CPC), cost per thousand impressions (CPM), and cost per view (CPV) for video content."

4b-i) Report if outcomes were (self-)assessed through online questionnaires

Clearly report if outcomes were (self-)assessed through online questionnaires (as common in web-based trials) or otherwise.

	1	2	3	4	5	
subitem not at all important	○	○	○	○	○	essential

Does your paper address subitem 4b-i? *

Copy and paste relevant sections from the manuscript (include quotes in quotation marks "like this" to indicate direct quotes from your manuscript), or elaborate on this item by providing additional information not in the ms, or briefly explain why the item is not applicable/relevant for your study

Not applicable. Outcomes were not assessed through online questionnaires.

4b-ii) Report how institutional affiliations are displayed

Report how institutional affiliations are displayed to potential participants [on ehealth media], as affiliations with prestigious hospitals or universities may affect volunteer rates, use, and reactions with regards to an intervention.(Not a required item – describe only if this may bias results)

	1	2	3	4	5	
subitem not at all important	○	○	○	○	○	essential

Does your paper address subitem 4b-ii?

Copy and paste relevant sections from the manuscript (include quotes in quotation marks "like this" to indicate direct quotes from your manuscript), or elaborate on this item by providing additional information not in the ms, or briefly explain why the item is not applicable/relevant for your study

Logos of the academic institution and community partners involved in developing the campaign were displayed at the bottom of the ads.

5) The interventions for each group with sufficient details to allow replication, including how and when they were actually administered

5-i) Mention names, credential, affiliations of the developers, sponsors, and owners

Mention names, credential, affiliations of the developers, sponsors, and owners [6] (if authors/evaluators are owners or developer of the software, this needs to be declared in a “Conflict of interest” section or mentioned elsewhere in the manuscript).

	1	2	3	4	5	
subitem not at all important	○	○	○	○	○	essential

Does your paper address subitem 5-i?

Copy and paste relevant sections from the manuscript (include quotes in quotation marks "like this" to indicate direct quotes from your manuscript), or elaborate on this item by providing additional information not in the ms, or briefly explain why the item is not applicable/relevant for your study

Your answer

5-ii) Describe the history/development process

Describe the history/development process of the application and previous formative evaluations (e.g., focus groups, usability testing), as these will have an impact on adoption/use rates and help with interpreting results.

	1	2	3	4	5	
subitem not at all important	○	○	○	○	○	essential

Does your paper address subitem 5-ii?

Copy and paste relevant sections from the manuscript (include quotes in quotation marks "like this" to indicate direct quotes from your manuscript), or elaborate on this item by providing additional information not in the ms, or briefly explain why the item is not applicable/relevant for your study

Your answer

5-iii) Revisions and updating

Revisions and updating. Clearly mention the date and/or version number of the application/intervention (and comparator, if applicable) evaluated, or describe whether the intervention underwent major changes during the evaluation process, or whether the development and/or content was “frozen” during the trial. Describe dynamic components such as news feeds or changing content which may have an impact on the replicability of the intervention (for unexpected events see item 3b).

	1	2	3	4	5	
subitem not at all important	○	○	○	○	○	essential

Does your paper address subitem 5-iii?

Copy and paste relevant sections from the manuscript (include quotes in quotation marks "like this" to indicate direct quotes from your manuscript), or elaborate on this item by providing additional information not in the ms, or briefly explain why the item is not applicable/relevant for your study

Your answer

5-iv) Quality assurance methods

Provide information on quality assurance methods to ensure accuracy and quality of information provided [1], if applicable.

	1	2	3	4	5	
subitem not at all important	○	○	○	○	○	essential

Does your paper address subitem 5-iv?

Copy and paste relevant sections from the manuscript (include quotes in quotation marks "like this" to indicate direct quotes from your manuscript), or elaborate on this item by providing additional information not in the ms, or briefly explain why the item is not applicable/relevant for your study

Your answer

5-v) Ensure replicability by publishing the source code, and/or providing screenshots/screen-capture video, and/or providing flowcharts of the algorithms used

Ensure replicability by publishing the source code, and/or providing screenshots/screen-capture video, and/or providing flowcharts of the algorithms used. Replicability (i.e., other researchers should in principle be able to replicate the study) is a hallmark of scientific reporting.

	1	2	3	4	5	
subitem not at all important	○	○	○	○	○	essential

Does your paper address subitem 5-v?

Copy and paste relevant sections from the manuscript (include quotes in quotation marks "like this" to indicate direct quotes from your manuscript), or elaborate on this item by providing additional information not in the ms, or briefly explain why the item is not applicable/relevant for your study

Your answer

5-vi) Digital preservation

Digital preservation: Provide the URL of the application, but as the intervention is likely to change or disappear over the course of the years; also make sure the intervention is archived (Internet Archive, webcitation.org, and/or publishing the source code or screenshots/videos alongside the article). As pages behind login screens cannot be archived, consider creating demo pages which are accessible without login.

	1	2	3	4	5	
subitem not at all important	○	○	○	○	○	essential

Does your paper address subitem 5-vi?

Copy and paste relevant sections from the manuscript (include quotes in quotation marks "like this" to indicate direct quotes from your manuscript), or elaborate on this item by providing additional information not in the ms, or briefly explain why the item is not applicable/relevant for your study

Your answer

5-vii) Access

Access: Describe how participants accessed the application, in what setting/context, if they had to pay (or were paid) or not, whether they had to be a member of specific group. If known, describe how participants obtained "access to the platform and Internet" [1]. To ensure access for editors/reviewers/readers, consider to provide a "backdoor" login account or demo mode for reviewers/readers to explore the application (also important for archiving purposes, see vi).

	1	2	3	4	5	
subitem not at all important	○	○	○	○	○	essential

Does your paper address subitem 5-vii? *

Copy and paste relevant sections from the manuscript (include quotes in quotation marks "like this" to indicate direct quotes from your manuscript), or elaborate on this item by providing additional information not in the ms, or briefly explain why the item is not applicable/relevant for your study

Individuals accessed the campaign through their usual use of the internet, apps, connected television, and digital radio.

"The campaign was placed through Google's Display & Video 360 platform (DV360), which delivers across more than 95 advertising exchanges and aggregates inventory from content websites, in-app placements, digital-radio networks (including audio versions of broadcast content), connected television apps, and YouTube. Unlike platform-native social media campaigns, DV360 does not place advertisements on Facebook, Instagram, or TikTok."

5-viii) Mode of delivery, features/functionalities/components of the intervention and comparator, and the theoretical framework

Describe mode of delivery, features/functionalities/components of the intervention and comparator, and the theoretical framework [6] used to design them (instructional strategy [1], behaviour change techniques, persuasive features, etc., see e.g., [7, 8] for terminology). This includes an in-depth description of the content (including where it is coming from and who developed it) [1]," whether [and how] it is tailored to individual circumstances and allows users to track their progress and receive feedback" [6]. This also includes a description of communication delivery channels and – if computer-mediated communication is a component – whether communication was synchronous or asynchronous [6]. It also includes information on presentation strategies [1], including page design principles, average amount of text on pages, presence of hyperlinks to other resources, etc. [1].

	1	2	3	4	5	
subitem not at all important	○	○	○	○	○	essential

Does your paper address subitem 5-viii? *

Copy and paste relevant sections from the manuscript (include quotes in quotation marks "like this" to indicate direct quotes from your manuscript), or elaborate on this item by providing additional information not in the ms, or briefly explain why the item is not applicable/relevant for your study

"The campaign was placed through Google's Display & Video 360 platform (DV360), which delivers across more than 95 advertising exchanges and aggregates inventory from content websites, in-app placements, digital-radio networks (including audio versions of broadcast content), connected television apps, and YouTube. Unlike platform-native social media campaigns, DV360 does not place advertisements on Facebook, Instagram, or TikTok. Placement of each impression was determined by DV360's automated bidder using a combination of explicit targeting parameters set by the campaign team and algorithmic optimization for the configured performance objective."

5-ix) Describe use parameters

Describe use parameters (e.g., intended "doses" and optimal timing for use). Clarify what instructions or recommendations were given to the user, e.g., regarding timing, frequency, heaviness of use, if any, or was the intervention used ad libitum.

	1	2	3	4	5	
subitem not at all important	○	○	○	○	○	essential

Does your paper address subitem 5-ix?

Copy and paste relevant sections from the manuscript (include quotes in quotation marks "like this" to indicate direct quotes from your manuscript), or elaborate on this item by providing additional information not in the ms, or briefly explain why the item is not applicable/relevant for your study

Your answer

5-x) Clarify the level of human involvement

Clarify the level of human involvement (care providers or health professionals, also technical assistance) in the e-intervention or as co-intervention (detail number and expertise of professionals involved, if any, as well as “type of assistance offered, the timing and frequency of the support, how it is initiated, and the medium by which the assistance is delivered”. It may be necessary to distinguish between the level of human involvement required for the trial, and the level of human involvement required for a routine application outside of a RCT setting (discuss under item 21 – generalizability).

	1	2	3	4	5	
subitem not at all important	○	○	○	○	○	essential

Does your paper address subitem 5-x?

Copy and paste relevant sections from the manuscript (include quotes in quotation marks "like this" to indicate direct quotes from your manuscript), or elaborate on this item by providing additional information not in the ms, or briefly explain why the item is not applicable/relevant for your study

Your answer

5-xi) Report any prompts/reminders used

Report any prompts/reminders used: Clarify if there were prompts (letters, emails, phone calls, SMS) to use the application, what triggered them, frequency etc. It may be necessary to distinguish between the level of prompts/reminders required for the trial, and the level of prompts/reminders for a routine application outside of a RCT setting (discuss under item 21 – generalizability).

	1	2	3	4	5	
subitem not at all important	○	○	○	○	○	essential

Does your paper address subitem 5-xi? *

Copy and paste relevant sections from the manuscript (include quotes in quotation marks "like this" to indicate direct quotes from your manuscript), or elaborate on this item by providing additional information not in the ms, or briefly explain why the item is not applicable/relevant for your study

There were no prompts or reminders to view or listen to ads. Using Google's Display & Video 360 platform, we set a "Frequency cap: twelve impressions per unique user across the campaign period."

5-xii) Describe any co-interventions (incl. training/support)

Describe any co-interventions (incl. training/support): Clearly state any interventions that are provided in addition to the targeted eHealth intervention, as ehealth intervention may not be designed as stand-alone intervention. This includes training sessions and support [1]. It may be necessary to distinguish between the level of training required for the trial, and the level of training for a routine application outside of a RCT setting (discuss under item 21 – generalizability.

	1	2	3	4	5	
subitem not at all important	○	○	○	○	○	essential

Does your paper address subitem 5-xii? *

Copy and paste relevant sections from the manuscript (include quotes in quotation marks "like this" to indicate direct quotes from your manuscript), or elaborate on this item by providing additional information not in the ms, or briefly explain why the item is not applicable/relevant for your study

Not applicable. This was an observational engagement study of a digital ad campaign served across internet, apps, connected television, and digital radio, not a randomized controlled trial.

6a) Completely defined pre-specified primary and secondary outcome measures, including how and when they were assessed

Does your paper address CONSORT subitem 6a? *

Copy and paste relevant sections from the manuscript (include quotes in quotation marks "like this" to indicate direct quotes from your manuscript), or elaborate on this item by providing additional information not in the ms, or briefly explain why the item is not applicable/relevant for your study

"Performance data were collected through DV360 and Google Analytics (GA) platforms using standardized digital marketing metrics. Key engagement metrics included impressions (the number of times ads were displayed to a user), reach (the total number of viewers), clicks (user interactions with ads), and click-through rate (CTR, calculated as clicks divided by impressions). For video content delivered through YouTube, connected television, and YouTube-engine partners, impressions were counted using Google's TrueView standard, which records a view only when a viewer watches at least 80% of the advertisement. Digital radio advertisements were counted as impressions when the audio played for any duration. Financial performance was tracked through cost per click (CPC), cost per thousand impressions (CPM), and cost per view (CPV) for video content.
Website engagement was monitored using several metrics, including traffic sources, bounce rates, average session duration, and pages per session. Language preference patterns were tracked to understand the effectiveness of bilingual content delivery. The platform also provided detailed information about device types and the geographic distribution of the audience."

6a-i) Online questionnaires: describe if they were validated for online use and apply CHERRIES items to describe how the questionnaires were designed/deployed

If outcomes were obtained through online questionnaires, describe if they were validated for online use and apply CHERRIES items to describe how the questionnaires were designed/deployed [9].

	1	2	3	4	5	
subitem not at all important	○	○	○	○	○	essential

Does your paper address subitem 6a-i?

Copy and paste relevant sections from manuscript text

Your answer

6a-ii) Describe whether and how “use” (including intensity of use/dosage) was defined/measured/monitored

Describe whether and how “use” (including intensity of use/dosage) was defined/measured/monitored (logins, logfile analysis, etc.). Use/adoption metrics are important process outcomes that should be reported in any ehealth trial.

	1	2	3	4	5	
subitem not at all important	○	○	○	○	○	essential

Does your paper address subitem 6a-ii?

Copy and paste relevant sections from manuscript text

Your answer

6a-iii) Describe whether, how, and when qualitative feedback from participants was obtained

Describe whether, how, and when qualitative feedback from participants was obtained (e.g., through emails, feedback forms, interviews, focus groups).

	1	2	3	4	5	
subitem not at all important	○	○	○	○	○	essential

Does your paper address subitem 6a-iii?

Copy and paste relevant sections from manuscript text

Your answer

6b) Any changes to trial outcomes after the trial commenced, with reasons

Does your paper address CONSORT subitem 6b? *

Copy and paste relevant sections from the manuscript (include quotes in quotation marks "like this" to indicate direct quotes from your manuscript), or elaborate on this item by providing additional information not in the ms, or briefly explain why the item is not applicable/relevant for your study

Not applicable. No changes were made to trial outcomes after the trial commenced.

7a) How sample size was determined

NPT: When applicable, details of whether and how the clustering by care provides or centers was addressed

7a-i) Describe whether and how expected attrition was taken into account when calculating the sample size

Describe whether and how expected attrition was taken into account when calculating the sample size.

	1	2	3	4	5	
subitem not at all important	○	○	○	○	○	essential

Does your paper address subitem 7a-i?

Copy and paste relevant sections from manuscript title (include quotes in quotation marks "like this" to indicate direct quotes from your manuscript), or elaborate on this item by providing additional information not in the ms, or briefly explain why the item is not applicable/relevant for your study

Your answer

7b) When applicable, explanation of any interim analyses and stopping guidelines

Does your paper address CONSORT subitem 7b? *

Copy and paste relevant sections from the manuscript (include quotes in quotation marks "like this" to indicate direct quotes from your manuscript), or elaborate on this item by providing additional information not in the ms, or briefly explain why the item is not applicable/relevant for your study

Not applicable. We completed the pilot intervention.

8a) Method used to generate the random allocation sequence

NPT: When applicable, how care providers were allocated to each trial group

Does your paper address CONSORT subitem 8a? *

Copy and paste relevant sections from the manuscript (include quotes in quotation marks "like this" to indicate direct quotes from your manuscript), or elaborate on this item by providing additional information not in the ms, or briefly explain why the item is not applicable/relevant for your study

Not applicable. This was an observational engagement study of a digital ad campaign served across internet, apps, connected television, and digital radio, not a randomized controlled trial.

8b) Type of randomisation; details of any restriction (such as blocking and block size)

Does your paper address CONSORT subitem 8b? *

Copy and paste relevant sections from the manuscript (include quotes in quotation marks "like this" to indicate direct quotes from your manuscript), or elaborate on this item by providing additional information not in the ms, or briefly explain why the item is not applicable/relevant for your study

Not applicable. This was an observational engagement study of a digital ad campaign served across internet, apps, connected television, and digital radio, not a randomized controlled trial.

9) Mechanism used to implement the random allocation sequence (such as sequentially numbered containers), describing any steps taken to conceal the sequence until interventions were assigned

Does your paper address CONSORT subitem 9? *

Copy and paste relevant sections from the manuscript (include quotes in quotation marks "like this" to indicate direct quotes from your manuscript), or elaborate on this item by providing additional information not in the ms, or briefly explain why the item is not applicable/relevant for your study

Not applicable. This was an observational engagement study of a digital ad campaign served across internet, apps, connected television, and digital radio, not a randomized controlled trial.

10) Who generated the random allocation sequence, who enrolled participants, and who assigned participants to interventions

Does your paper address CONSORT subitem 10? *

Copy and paste relevant sections from the manuscript (include quotes in quotation marks "like this" to indicate direct quotes from your manuscript), or elaborate on this item by providing additional information not in the ms, or briefly explain why the item is not applicable/relevant for your study

Not applicable. This was an observational engagement study of a digital ad campaign served across internet, apps, connected television, and digital radio, not a randomized controlled trial.

11a) If done, who was blinded after assignment to interventions (for example, participants, care providers, those assessing outcomes) and how

NPT: Whether or not administering co-interventions were blinded to group assignment

11a-i) Specify who was blinded, and who wasn't

Specify who was blinded, and who wasn't. Usually, in web-based trials it is not possible to blind the participants [1, 3] (this should be clearly acknowledged), but it may be possible to blind outcome assessors, those doing data analysis or those administering co-interventions (if any).

	1	2	3	4	5	
subitem not at all important	○	○	○	○	○	essential

Does your paper address subitem 11a-i? *

Copy and paste relevant sections from the manuscript (include quotes in quotation marks "like this" to indicate direct quotes from your manuscript), or elaborate on this item by providing additional information not in the ms, or briefly explain why the item is not applicable/relevant for your study

No individuals involved in this observational study were blinded.

11a-ii) Discuss e.g., whether participants knew which intervention was the "intervention of interest" and which one was the "comparator"

Informed consent procedures (4a-ii) can create biases and certain expectations - discuss e.g., whether participants knew which intervention was the "intervention of interest" and which one was the "comparator".

	1	2	3	4	5	
subitem not at all important	○	○	○	○	○	essential

Does your paper address subitem 11a-ii?

Copy and paste relevant sections from the manuscript (include quotes in quotation marks "like this" to indicate direct quotes from your manuscript), or elaborate on this item by providing additional information not in the ms, or briefly explain why the item is not applicable/relevant for your study

Your answer

11b) If relevant, description of the similarity of interventions

(this item is usually not relevant for ehealth trials as it refers to similarity of a placebo or sham intervention to a active medication/intervention)

Does your paper address CONSORT subitem 11b? *

Copy and paste relevant sections from the manuscript (include quotes in quotation marks "like this" to indicate direct quotes from your manuscript), or elaborate on this item by providing additional information not in the ms, or briefly explain why the item is not applicable/relevant for your study

Not applicable. This was an observational engagement study of a digital ad campaign served across internet, apps, connected television, and digital radio, not a randomized controlled trial.

12a) Statistical methods used to compare groups for primary and secondary outcomes
NPT: When applicable, details of whether and how the clustering by care providers or centers was addressed

Does your paper address CONSORT subitem 12a? *

Copy and paste relevant sections from the manuscript (include quotes in quotation marks "like this" to indicate direct quotes from your manuscript), or elaborate on this item by providing additional information not in the ms, or briefly explain why the item is not applicable/relevant for your study

"Performance metrics — impressions, clicks, CTR, CPC, CPM, and CPV — were extracted from DV360 monthly per-creative exports and aggregated to the format × language × topic level (Table 1) and to the line-item level for device-specific analyses (Table 3). Click-through rates are reported with Wilson 95% confidence intervals to account for the small denominators in the audio cells.
For the substantively important comparisons, we report two-proportion z-tests (equivalent to Pearson χ^2 on the 2×2 table of clicks and non-clicks) at the platform-aggregate level: (a) Vietnamese versus English display CTR; (b) display versus video CTR; and (c) audio versus display CTR. We treat these as descriptive comparisons of platform-level aggregates rather than tests of individual-level hypotheses, because impressions are not independent user observations (the same user may receive multiple impressions under the frequency cap of twelve) and because format-level comparisons are confounded with phase, audience-segment targeting, and the platform's automated bidder. Effect sizes are reported as risk ratios alongside p-values.
We did not perform parametric tests (t-tests or ANOVA) on monthly CTR observations within format. With two to three monthly observations per medium and confounding between format and phase, parametric inference at the medium-month level is not adequately powered. The reported month-level CTR variation is presented descriptively. Website-engagement metrics from Google Analytics — sessions, language distribution, bounce rate, session duration, and pages per session — are tabulated by campaign phase and a pre-campaign baseline window. Phase 2 landing-page distribution by language was inferred from Google Analytics Behavior Flow reporting because each ad pointed to a specific language-distinguished landing URL."

12a-i) Imputation techniques to deal with attrition / missing values

Imputation techniques to deal with attrition / missing values: Not all participants will use the intervention/comparator as intended and attrition is typically high in ehealth trials. Specify how participants who did not use the application or dropped out from the trial were treated in the statistical analysis (a complete case analysis is strongly discouraged, and simple imputation techniques such as LOCF may also be problematic [4]).

	1	2	3	4	5	
subitem not at all important	○	○	○	○	○	essential

Does your paper address subitem 12a-i? *

Copy and paste relevant sections from the manuscript (include quotes in quotation marks "like this" to indicate direct quotes from your manuscript), or elaborate on this item by providing additional information not in the ms, or briefly explain why the item is not applicable/relevant for your study

Not applicable. This was an observational engagement study of a digital ad campaign served across internet, apps, connected television, and digital radio, not a randomized controlled trial.

12b) Methods for additional analyses, such as subgroup analyses and adjusted analyses

Does your paper address CONSORT subitem 12b? *

Copy and paste relevant sections from the manuscript (include quotes in quotation marks "like this" to indicate direct quotes from your manuscript), or elaborate on this item by providing additional information not in the ms, or briefly explain why the item is not applicable/relevant for your study

Not applicable. "Performance metrics — impressions, clicks, CTR, CPC, CPM, and CPV — were extracted from DV360 monthly per-creative exports and aggregated to the format × language × topic level (Table 1) and to the line-item level for device-specific analyses (Table 3). Click-through rates are reported with Wilson 95% confidence intervals to account for the small denominators in the audio cells.

For the substantively important comparisons, we report two-proportion z-tests (equivalent to Pearson χ^2 on the 2×2 table of clicks and non-clicks) at the platform-aggregate level: (a) Vietnamese versus English display CTR; (b) display versus video CTR; and (c) audio versus display CTR. We treat these as descriptive comparisons of platform-level aggregates rather than tests of individual-level hypotheses, because impressions are not independent user observations (the same user may receive multiple impressions under the frequency cap of twelve) and because format-level comparisons are confounded with phase, audience-segment targeting, and the platform's automated bidder. Effect sizes are reported as risk ratios alongside p-values.

We did not perform parametric tests (t-tests or ANOVA) on monthly CTR observations within format. With two to three monthly observations per medium and confounding between format and phase, parametric inference at the medium-month level is not adequately powered. The reported month-level CTR variation is presented descriptively. Website-engagement metrics from Google Analytics — sessions, language distribution, bounce rate, session duration, and pages per session — are tabulated by campaign phase and a pre-campaign baseline window. Phase 2 landing-page distribution by language was inferred from Google Analytics Behavior Flow reporting because each ad pointed to a specific language-distinguished landing URL."

X26) REB/IRB Approval and Ethical Considerations [recommended as subheading under "Methods"] (not a CONSORT item)

X26-i) Comment on ethics committee approval

	1	2	3	4	5	
subitem not at all important	○	○	○	○	○	essential

Does your paper address subitem X26-i?

Copy and paste relevant sections from the manuscript (include quotes in quotation marks "like this" to indicate direct quotes from your manuscript), or elaborate on this item by providing additional information not in the ms, or briefly explain why the item is not applicable/relevant for your study

Your answer

x26-ii) Outline informed consent procedures

Outline informed consent procedures e.g., if consent was obtained offline or online (how? Checkbox, etc.?), and what information was provided (see 4a-ii). See [6] for some items to be included in informed consent documents.

	1	2	3	4	5	
subitem not at all important	○	○	○	○	○	essential

Does your paper address subitem X26-ii?

Copy and paste relevant sections from the manuscript (include quotes in quotation marks "like this" to indicate direct quotes from your manuscript), or elaborate on this item by providing additional information not in the ms, or briefly explain why the item is not applicable/relevant for your study

Your answer

X26-iii) Safety and security procedures

Safety and security procedures, incl. privacy considerations, and any steps taken to reduce the likelihood or detection of harm (e.g., education and training, availability of a hotline)

	1	2	3	4	5	
subitem not at all important	○	○	○	○	○	essential

Does your paper address subitem X26-iii?

Copy and paste relevant sections from the manuscript (include quotes in quotation marks "like this" to indicate direct quotes from your manuscript), or elaborate on this item by providing additional information not in the ms, or briefly explain why the item is not applicable/relevant for your study

Your answer

RESULTS

13a) For each group, the numbers of participants who were randomly assigned, received intended treatment, and were analysed for the primary outcome

NPT: The number of care providers or centers performing the intervention in each group and the number of patients treated by each care provider in each center

Does your paper address CONSORT subitem 13a? *

Copy and paste relevant sections from the manuscript (include quotes in quotation marks "like this" to indicate direct quotes from your manuscript), or elaborate on this item by providing additional information not in the ms, or briefly explain why the item is not applicable/relevant for your study

Not applicable. This was an observational engagement study of a digital ad campaign served across internet, apps, connected television, and digital radio, not a randomized controlled trial.

13b) For each group, losses and exclusions after randomisation, together with reasons

Does your paper address CONSORT subitem 13b? (NOTE: Preferably, this is shown in a CONSORT flow diagram) *

Copy and paste relevant sections from the manuscript (include quotes in quotation marks "like this" to indicate direct quotes from your manuscript), or elaborate on this item by providing additional information not in the ms, or briefly explain why the item is not applicable/relevant for your study

Not applicable. This was an observational engagement study of a digital ad campaign served across internet, apps, connected television, and digital radio, not a randomized controlled trial.

13b-i) Attrition diagram

Strongly recommended: An attrition diagram (e.g., proportion of participants still logging in or using the intervention/comparator in each group plotted over time, similar to a survival curve) or other figures or tables demonstrating usage/dose/engagement.

	1	2	3	4	5	
subitem not at all important	○	○	○	○	○	essential

Does your paper address subitem 13b-i?

Copy and paste relevant sections from the manuscript or cite the figure number if applicable (include quotes in quotation marks "like this" to indicate direct quotes from your manuscript), or elaborate on this item by providing additional information not in the ms, or briefly explain why the item is not applicable/relevant for your study

Your answer

14a) Dates defining the periods of recruitment and follow-up

Does your paper address CONSORT subitem 14a? *

Copy and paste relevant sections from the manuscript (include quotes in quotation marks "like this" to indicate direct quotes from your manuscript), or elaborate on this item by providing additional information not in the ms, or briefly explain why the item is not applicable/relevant for your study

Not applicable. This was an observational engagement study of a digital ad campaign served across internet, apps, connected television, and digital radio, not a randomized controlled trial.

14a-i) Indicate if critical "secular events" fell into the study period

Indicate if critical "secular events" fell into the study period, e.g., significant changes in Internet resources available or "changes in computer hardware or Internet delivery resources"

	1	2	3	4	5	
subitem not at all important	○	○	○	○	○	essential

Does your paper address subitem 14a-i?

Copy and paste relevant sections from the manuscript (include quotes in quotation marks "like this" to indicate direct quotes from your manuscript), or elaborate on this item by providing additional information not in the ms, or briefly explain why the item is not applicable/relevant for your study

Your answer

14b) Why the trial ended or was stopped (early)

Does your paper address CONSORT subitem 14b? *

Copy and paste relevant sections from the manuscript (include quotes in quotation marks "like this" to indicate direct quotes from your manuscript), or elaborate on this item by providing additional information not in the ms, or briefly explain why the item is not applicable/relevant for your study

The trial ended at the end of the grant funding period.

15) A table showing baseline demographic and clinical characteristics for each group

NPT: When applicable, a description of care providers (case volume, qualification, expertise, etc.) and centers (volume) in each group

Does your paper address CONSORT subitem 15? *

Copy and paste relevant sections from the manuscript (include quotes in quotation marks "like this" to indicate direct quotes from your manuscript), or elaborate on this item by providing additional information not in the ms, or briefly explain why the item is not applicable/relevant for your study

Because this was an observational engagement study of a digital ad campaign served across internet, apps, connected television, and digital radio, we did not have a way to capture unique individual demographics.

15-i) Report demographics associated with digital divide issues

In ehealth trials it is particularly important to report demographics associated with digital divide issues, such as age, education, gender, social-economic status, computer/Internet/ ehealth literacy of the participants, if known.

	1	2	3	4	5	
subitem not at all important	○	○	○	○	○	essential

Does your paper address subitem 15-i? *

Copy and paste relevant sections from the manuscript (include quotes in quotation marks "like this" to indicate direct quotes from your manuscript), or elaborate on this item by providing additional information not in the ms, or briefly explain why the item is not applicable/relevant for your study

Because this was an observational engagement study of a digital ad campaign served across internet, apps, connected television, and digital radio, we did not have a way to capture unique individual demographics.

16) For each group, number of participants (denominator) included in each analysis and whether the analysis was by original assigned groups

16-i) Report multiple "denominators" and provide definitions

Report multiple "denominators" and provide definitions: Report N's (and effect sizes) "across a range of study participation [and use] thresholds" [1], e.g., N exposed, N consented, N used more than x times, N used more than y weeks, N participants "used" the intervention/comparator at specific pre-defined time points of interest (in absolute and relative numbers per group). Always clearly define "use" of the intervention.

	1	2	3	4	5	
subitem not at all important	○	○	○	○	○	essential

Does your paper address subitem 16-i? *

Copy and paste relevant sections from the manuscript (include quotes in quotation marks "like this" to indicate direct quotes from your manuscript), or elaborate on this item by providing additional information not in the ms, or briefly explain why the item is not applicable/relevant for your study

"The digital health campaign achieved substantial audience exposure, generating 4,974,357 classifiable impressions across all formats over a 19-week period plus 304,424 unclassified June 2022 impressions reported separately under Cross-Platform Engagement. The campaign reached approximately 1.4 million unique users during the display phase (frequency 2.8 ad views per person) and approximately 105,000 unique users during the audiovisual phase (frequency 3.06 ad views per person; Supplementary File S8 — DV360 reach summary). Impressions reflect ad delivery within geographic targeting (Texas) and platform-side language, contextual, and audience-segment signals; the proportion of impressions actually served to Vietnamese American users is not directly measurable from platform-side reporting."

16-ii) Primary analysis should be intent-to-treat

Primary analysis should be intent-to-treat, secondary analyses could include comparing only "users", with the appropriate caveats that this is no longer a randomized sample (see 18-i).

	1	2	3	4	5	
subitem not at all important	○	○	○	○	○	essential

Does your paper address subitem 16-ii?

Copy and paste relevant sections from the manuscript (include quotes in quotation marks "like this" to indicate direct quotes from your manuscript), or elaborate on this item by providing additional information not in the ms, or briefly explain why the item is not applicable/relevant for your study

Your answer

17a) For each primary and secondary outcome, results for each group, and the estimated effect size and its precision (such as 95% confidence interval)

Does your paper address CONSORT subitem 17a? *

Copy and paste relevant sections from the manuscript (include quotes in quotation marks "like this" to indicate direct quotes from your manuscript), or elaborate on this item by providing additional information not in the ms, or briefly explain why the item is not applicable/relevant for your study

Table 1 of the manuscript contains "Campaign Performance Metrics by Format, Language, and Topic."

17a-i) Presentation of process outcomes such as metrics of use and intensity of use

In addition to primary/secondary (clinical) outcomes, the presentation of process outcomes such as metrics of use and intensity of use (dose, exposure) and their operational definitions is critical. This does not only refer to metrics of attrition (13-b) (often a binary variable), but also to more continuous exposure metrics such as "average session length". These must be accompanied by a technical description how a metric like a "session" is defined (e.g., timeout after idle time) [1] (report under item 6a).

	1	2	3	4	5	
subitem not at all important	○	○	○	○	○	essential

Does your paper address subitem 17a-i?

Copy and paste relevant sections from the manuscript (include quotes in quotation marks "like this" to indicate direct quotes from your manuscript), or elaborate on this item by providing additional information not in the ms, or briefly explain why the item is not applicable/relevant for your study

Your answer

17b) For binary outcomes, presentation of both absolute and relative effect sizes is recommended

Does your paper address CONSORT subitem 17b? *

Copy and paste relevant sections from the manuscript (include quotes in quotation marks "like this" to indicate direct quotes from your manuscript), or elaborate on this item by providing additional information not in the ms, or briefly explain why the item is not applicable/relevant for your study

"Performance of Static Display Advertisements
English-language static advertisements achieved 2,446,027 impressions; Vietnamese-language counterparts generated 1,533,007 impressions. Performance varied by display dimension and placement; per-dimension impression counts are reported in Supplementary Table S8.
Performance of Audiovisual Advertisements
Video advertisement impressions ranged from 170,757 to 330,579 across the four language-topic cells. The Vietnamese-language vaccination video achieved the highest engagement (330,579 impressions; CTR 0.051%), followed by the English vaccination video (247,387 impressions; CTR 0.028%), English clinical trial video (244,766 impressions; CTR 0.033%), and Vietnamese clinical trial video (170,757 impressions; CTR 0.048%).
The audio component reached a smaller audience. Audio advertisements about COVID-19 vaccination narrated in English received 262 impressions and Vietnamese 633 impressions; clinical-trial audio impressions were 297 (English) and 642 (Vietnamese). Across all four audio cells, 5 clicks were observed on 1,834 impressions.
Platform and Device Engagement
Analysis of device-attributed video, audio, and YouTube impressions revealed that mobile phones dominated content delivery, generating 608,428 impressions (61.2% of device-attributed impressions). Desktop computers followed with 338,370 impressions (34.1%), while tablets and connected-television devices accounted for 46,557 and 134 impressions, respectively (Table 2). Static-display impressions are not device-segmented in DV360's per-creative export at the same granularity, and the proportions reported in Table 2 should be interpreted as device-attributed video/CTV/audio impressions rather than total reach.
Engagement and Cost Efficiency Metrics
The campaign's overall CTR was 0.064%. Cost efficiency was evaluated using multiple metrics. The average CPC across the campaign was $2.35, with individual advertisements ranging from $0.03 to $9.95. The CPM averaged $1.45 (range $0.70–$5.12). The video content CPV averaged $2.31, with minimal variation ($2.19–$2.51).
Temporal Patterns and Inferential Comparisons
Aggregated monthly click-through rates differed substantially within format. Standard display CTRs were stable at 0.066–0.074% across February and March 2022 and 0.057% in June. Video CTR was stable at 0.040% in both May and June. Audio CTR was 1.911% in May 2022, falling to 0.119% in June — a sixteen-fold drop on a small impression base. The May audio peak rests on approximately 5 clicks across 262 English-audio impressions and a 95% Wilson confidence interval of 0.07–2.13%; the rate did not replicate in June, and we interpret the audio observation cautiously.
Two-proportion comparisons across format–language cells confirm three differences. First, Vietnamese display achieved a higher CTR than English display ($\chi^2 = 480.6$, $p < .001$; risk ratio 2.29). Second, display as a class achieved a higher CTR than video ($\chi^2 = 102.6$, $p < .001$). Third, audio CTR exceeded display CTR even under the small-N caveat ($\chi^2 = 11.1$, $p = .001$), but rests on only five observed audio clicks; the result is statistically present but fragile, and we treat it as hypothesis-generating rather than evidence of durable audio superiority. Within the display arm, mobile placements achieved approximately three-fold higher CTR than desktop placements within each language (Table 3); the device-stratified impression split for display is not available in the per-creative export, so a parallel inferential test for device is not reported here.
Audience Geography and Phase 2 Landing-Page Distribution

Impressions accumulated in expected Texas metropolitan areas with substantial Vietnamese American populations. The five largest cities — Houston, Dallas, Austin, San Antonio, and Fort Worth — accounted for approximately 60% of total impressions; together with the next twelve Texas cities they accounted for over 80% (Table 4; full city-level distribution in Supplementary Table S6). Approximately 4.7% of impressions were attributed to an "unknown" city. DV360 reports did not provide age or gender breakdowns at the user level; we therefore do not report age/gender stability across phases.

During Phase 2 (May 26 – June 30, 2022), Google Analytics Behavior Flow reporting captured 717 sessions with identifiable landing-page URLs. Of the 612 sessions that landed on a language-distinguished campaign page (excluding 105 sessions that landed on the homepage or an unlabeled page), 338 (55.2%) landed on a Vietnamese-language page (e.g., /thong-tin-ve-covid-19-va-vac-xin/...) and 274 (44.8%) landed on an English-language page (/vaccine-info/...). The deviation from a 50:50 split favored Vietnamese-language pages (χ^2 = 6.7, df = 1, p = .010); the Vietnamese vaccine-information landing page received the largest single-page share (n = 225; 31.4% of Phase 2 sessions). Landing-page language is distinct from user browser language: our Table 5 reports browser language (21.9% Vietnamese in Phase 2), capturing a different signal, while the Behavior Flow data indicate that Phase 2 ad-driven traffic was directed to Vietnamese-language content for the majority of clickers regardless of their browser-side language settings (Supplementary Table S9).

Of all 717 Phase 2 Behavior Flow sessions, 673 (93.9%) ended at the landing page without progressing to a second page; 44 sessions (6.1%) progressed to a first interaction, and 18 (2.5%) reached a second interaction. The high single-page rate is comparable to industry rates for digital-display-driven traffic and reflects the brevity of typical ad-click sessions.

Website Engagement Analysis

The campaign drove traffic to the VietCOVID website, with substantial increases during both campaign phases (Table 5). During the static display campaign, website visitors increased from 270 (pre-display baseline) to 6,437, representing a 23.84-fold increase in traffic. The audiovisual campaign period saw an increase from 133 to 780 visitors.

Language preference of website sessions, as captured by user browser settings, differed between phases. During the display phase, English-detected sessions comprised 93.48% of traffic and Vietnamese-detected sessions 6.07%. The audiovisual phase showed a shift, with English-detected sessions at 73.91% and Vietnamese-detected sessions rising to 21.85%. As noted under Audience Geography and Phase 2 Landing-Page Distribution, ad-driven landing-page language in Phase 2 was majority-Vietnamese even though browser-detected language remained majority-English."

18) Results of any other analyses performed, including subgroup analyses and adjusted analyses, distinguishing pre-specified from exploratory

Does your paper address CONSORT subitem 18? *

Copy and paste relevant sections from the manuscript (include quotes in quotation marks "like this" to indicate direct quotes from your manuscript), or elaborate on this item by providing additional information not in the ms, or briefly explain why the item is not applicable/relevant for your study

Not applicable. Exploratory subgroup analyses were not performed.

18-i) Subgroup analysis of comparing only users

A subgroup analysis of comparing only users is not uncommon in ehealth trials, but if done, it must be stressed that this is a self-selected sample and no longer an unbiased sample from a randomized trial (see 16-iii).

	1	2	3	4	5	
subitem not at all important	○	○	○	○	○	essential

Does your paper address subitem 18-i?

Copy and paste relevant sections from the manuscript (include quotes in quotation marks "like this" to indicate direct quotes from your manuscript), or elaborate on this item by providing additional information not in the ms, or briefly explain why the item is not applicable/relevant for your study

Your answer

19) All important harms or unintended effects in each group

(for specific guidance see CONSORT for harms)

Does your paper address CONSORT subitem 19? *

Copy and paste relevant sections from the manuscript (include quotes in quotation marks "like this" to indicate direct quotes from your manuscript), or elaborate on this item by providing additional information not in the ms, or briefly explain why the item is not applicable/relevant for your study

Not applicable. This was an observational engagement study of a digital ad campaign served across internet, apps, connected television, and digital radio, not a randomized controlled trial.

19-i) Include privacy breaches, technical problems

Include privacy breaches, technical problems. This does not only include physical "harm" to participants, but also incidents such as perceived or real privacy breaches [1], technical problems, and other unexpected/unintended incidents. "Unintended effects" also includes unintended positive effects [2].

	1	2	3	4	5	
subitem not at all important	○	○	○	○	○	essential

Does your paper address subitem 19-i?

Copy and paste relevant sections from the manuscript (include quotes in quotation marks "like this" to indicate direct quotes from your manuscript), or elaborate on this item by providing additional information not in the ms, or briefly explain why the item is not applicable/relevant for your study

Your answer

19-ii) Include qualitative feedback from participants or observations from staff/ researchers

Include qualitative feedback from participants or observations from staff/researchers, if available, on strengths and shortcomings of the application, especially if they point to unintended/unexpected effects or uses. This includes (if available) reasons for why people did or did not use the application as intended by the developers.

	1	2	3	4	5	
subitem not at all important	○	○	○	○	○	essential

Does your paper address subitem 19-ii?

Copy and paste relevant sections from the manuscript (include quotes in quotation marks "like this" to indicate direct quotes from your manuscript), or elaborate on this item by providing additional information not in the ms, or briefly explain why the item is not applicable/relevant for your study

Your answer

DISCUSSION

22) Interpretation consistent with results, balancing benefits and harms, and considering other relevant evidence

NPT: In addition, take into account the choice of the comparator, lack of or partial blinding, and unequal expertise of care providers or centers in each group

22-i) Restate study questions and summarize the answers suggested by the data, starting with primary outcomes and process outcomes (use)

Restate study questions and summarize the answers suggested by the data, starting with primary outcomes and process outcomes (use).

	1	2	3	4	5	
subitem not at all important	○	○	○	○	○	essential

Does your paper address subitem 22-i? *

Copy and paste relevant sections from the manuscript (include quotes in quotation marks "like this" to indicate direct quotes from your manuscript), or elaborate on this item by providing additional information not in the ms, or briefly explain why the item is not applicable/relevant for your study

"We implemented a bilingual digital health campaign for Vietnamese Americans in Texas, delivered through Google's Display & Video 360 platform across static display, video, audio, YouTube, and connected-television inventory between February and June 2022, with a total budget of $9,472.54. The campaign delivered approximately 5 million impressions to roughly 1.4 million unique users in Phase 1 and 105,000 unique users in Phase 2, and was associated with substantial increases in traffic to the campaign's information website during both phases. We did not measure downstream vaccination uptake or clinical-trial enrollment; the outcomes reported are exposure and engagement metrics only, and our findings should not be read as evidence of behavior change [14,19,20].
Three engagement findings are noteworthy. First, within static display, Vietnamese-language ads outperformed English-language ads on a click-through basis (Vietnamese display CTR 0.105%, 95% CI 0.100–0.110% vs. English display CTR 0.046%, 95% CI 0.043–0.049%; χ^2 = 480.6, $p < .001$; risk ratio 2.29). Vietnamese display also generated more clicks in absolute terms (1,610 vs. 1,122) despite reaching approximately 37% fewer impressions. Second, within each language, mobile placements outperformed desktop placements by roughly threefold at the line-item level (Vietnamese mobile 0.156% vs. Vietnamese desktop 0.045%; English mobile 0.064% vs. English desktop 0.019%; Table 3). Third, audio click-through was high in May 2022 (1.911%) but fell sixteen-fold to 0.119% in June 2022, with broad confidence intervals reflecting small audio impression denominators. At the platform-aggregate level, audio CTR was statistically higher than display CTR (χ^2 = 11.1, $p = .001$), but the test rests on only five observed audio clicks and is sensitive to small variations in count. The audio finding is therefore hypothesis-generating rather than evidence of durable audio superiority."

22-ii) Highlight unanswered new questions, suggest future research

Highlight unanswered new questions, suggest future research.

	1	2	3	4	5	
subitem not at all important	○	○	○	○	○	essential

Does your paper address subitem 22-ii?

Copy and paste relevant sections from the manuscript (include quotes in quotation marks "like this" to indicate direct quotes from your manuscript), or elaborate on this item by providing additional information not in the ms, or briefly explain why the item is not applicable/relevant for your study

Your answer

20) Trial limitations, addressing sources of potential bias, imprecision, and, if relevant, multiplicity of analyses

20-i) Typical limitations in ehealth trials

Typical limitations in ehealth trials: Participants in ehealth trials are rarely blinded. Ehealth trials often look at a multiplicity of outcomes, increasing risk for a Type I error. Discuss biases due to non-use of the intervention/usability issues, biases through informed consent procedures, unexpected events.

	1	2	3	4	5	
subitem not at all important	○	○	○	○	○	essential

Does your paper address subitem 20-i? *

Copy and paste relevant sections from the manuscript (include quotes in quotation marks "like this" to indicate direct quotes from your manuscript), or elaborate on this item by providing additional information not in the ms, or briefly explain why the item is not applicable/relevant for your study

"Several limitations warrant consideration. First, DV360 does not verify user ethnicity, age, or gender; reach to Vietnamese American users is inferred from language-and-locale signals, contextual placement, and Phase 2 audience-segment targeting. Because click-through is measured on consistent ad content, ethnic misclassification of English-arm clickers should attenuate rather than inflate any Vietnamese American–specific effect, biasing English-arm estimates toward the null. The audience-stability claim is therefore restricted to geographic distribution and language settings.
Second, sequential phasing and opaque automated placement confound format comparisons. Display ran in February–March and audiovisual in May–June, and DV360's bidder selected specific apps and time slots without per-impression transparency. The design supports comparative description within each phase but not causal attribution to format alone; the top-app analysis (Supplementary Tables S4–S5) is the strongest available placement transparency.
Third, the audio impression base is small (≈1,800 total) and audio CTRs have correspondingly wide confidence intervals; the May 2022 peak should not be over-interpreted.
Fourth, no post-engagement behavioral data linked clicks to vaccination or trial enrolment. Future campaigns could bridge this gap via campaign-unique landing URLs with opt-in handoff to a partner clinic's electronic health record (EHR) system, brief on-page intake of vaccination status or intent, or zip-code-level linkage to public-health vaccination dashboards. Our group's parallel cohort work shows that digital-only engagement strategies under-represent primary Vietnamese speakers, female respondents, and individuals without a college degree [19]; platform-side metrics therefore likely understate reach to the most vulnerable subpopulations.
Fifth, the revised ads were not pilot-tested before the February 15 launch; the focus-group review with BPSOSH was the only pre-launch validation.
Sixth, digital ad platforms remain vulnerable to the broader infodemic[1]. The Vietnamese American diaspora's use of encrypted apps such as Zalo for intergenerational health-information sharing is largely opaque to platform-level outreach; targeted disinformation — including coordinated narratives circulated through diaspora-specific networks — further undermines platform-only approaches[31]. Complementary grassroots and trusted-messenger strategies, including infodemiology-informed dynamic content adaptation, remain necessary [30-32]."

21) Generalisability (external validity, applicability) of the trial findings

NPT: External validity of the trial findings according to the intervention, comparators, patients, and care providers or centers involved in the trial

21-i) Generalizability to other populations

Generalizability to other populations: In particular, discuss generalizability to a general Internet population, outside of a RCT setting, and general patient population, including applicability of the study results for other organizations

	1	2	3	4	5	
subitem not at all important	○	○	○	○	○	essential

Does your paper address subitem 21-i?

Copy and paste relevant sections from the manuscript (include quotes in quotation marks "like this" to indicate direct quotes from your manuscript), or elaborate on this item by providing additional information not in the ms, or briefly explain why the item is not applicable/relevant for your study

Your answer

21-ii) Discuss if there were elements in the RCT that would be different in a routine application setting

Discuss if there were elements in the RCT that would be different in a routine application setting (e.g., prompts/reminders, more human involvement, training sessions or other co-interventions) and what impact the omission of these elements could have on use, adoption, or outcomes if the intervention is applied outside of a RCT setting.

	1	2	3	4	5	
subitem not at all important	○	○	○	○	○	essential

Does your paper address subitem 21-ii?

Copy and paste relevant sections from the manuscript (include quotes in quotation marks "like this" to indicate direct quotes from your manuscript), or elaborate on this item by providing additional information not in the ms, or briefly explain why the item is not applicable/relevant for your study

Your answer

OTHER INFORMATION

23) Registration number and name of trial registry

Does your paper address CONSORT subitem 23? *

Copy and paste relevant sections from the manuscript (include quotes in quotation marks "like this" to indicate direct quotes from your manuscript), or elaborate on this item by providing additional information not in the ms, or briefly explain why the item is not applicable/relevant for your study

Not applicable. This was an observational engagement study of a digital ad campaign served across internet, apps, connected television, and digital radio, not a randomized controlled trial.

24) Where the full trial protocol can be accessed, if available

Does your paper address CONSORT subitem 24? *

Cite a Multimedia Appendix, other reference, or copy and paste relevant sections from the manuscript (include quotes in quotation marks "like this" to indicate direct quotes from your manuscript), or elaborate on this item by providing additional information not in the ms, or briefly explain why the item is not applicable/relevant for your study

Not applicable. This was an observational engagement study of a digital ad campaign served across internet, apps, connected television, and digital radio, not a randomized controlled trial.

25) Sources of funding and other support (such as supply of drugs), role of funders

Does your paper address CONSORT subitem 25? *

Copy and paste relevant sections from the manuscript (include quotes in quotation marks "like this" to indicate direct quotes from your manuscript), or elaborate on this item by providing additional information not in the ms, or briefly explain why the item is not applicable/relevant for your study

"This research was, in part, partly funded by the National Institutes of Health (NIH) Agreement OT2HL158287.
Disclaimer: The views and conclusions in this document are those of the authors and should not be interpreted as representing the official policies, either expressed or implied, of the NIH."

X27) Conflicts of Interest (not a CONSORT item)

X27-i) State the relation of the study team towards the system being evaluated

In addition to the usual declaration of interests (financial or otherwise), also state the relation of the study team towards the system being evaluated, i.e., state if the authors/evaluators are distinct from or identical with the developers/sponsors of the intervention.

	1	2	3	4	5	
subitem not at all important	○	○	○	○	○	essential

Does your paper address subitem X27-i?

Copy and paste relevant sections from the manuscript (include quotes in quotation marks "like this" to indicate direct quotes from your manuscript), or elaborate on this item by providing additional information not in the ms, or briefly explain why the item is not applicable/relevant for your study

Your answer

About the CONSORT EHEALTH checklist

As a result of using this checklist, did you make changes in your manuscript? *

- ○ yes, major changes
- ◉ yes, minor changes
- ○ no

What were the most important changes you made as a result of using this checklist?

Your answer

How much time did you spend on going through the checklist INCLUDING making changes in your manuscript *

It took me about 2 hours to review the checklist and update the manuscript.

As a result of using this checklist, do you think your manuscript has improved? *

- (•) yes
- ( ) no
- ( ) Other:

Would you like to become involved in the CONSORT EHEALTH group?

This would involve for example becoming involved in participating in a workshop and writing an "Explanation and Elaboration" document

- ( ) yes
- (•) no
- ( ) Other:

Clear selection

Any other comments or questions on CONSORT EHEALTH

Your answer

STOP - Save this form as PDF before you click submit

To generate a record that you filled in this form, we recommend to generate a PDF of this page (on a Mac, simply select "print" and then select "print as PDF") before you submit it.

When you submit your (revised) paper to JMIR, please upload the PDF as supplementary file.

Don't worry if some text in the textboxes is cut off, as we still have the complete information in our database. Thank you!

Final step: Click submit !

Click submit so we have your answers in our database!

Submit

Clear form

Never submit passwords through Google Forms.

This content is neither created nor endorsed by Google. - Terms of Service - Privacy Policy

Does this form look suspicious? Report

Google Forms
