# Supplementary material for: Digital Campaign to Promote COVID-19 Vaccination and Clinical Trial Participation Among Vietnamese Americans: Observational Engagement Study
Source: J Med Internet Res. 2026 Oct 15;28:e90301. doi: 10.2196/90301 (PMC13689936; doi:10.2196/90301)
Supplement: Supplementary file 2 [file jmir-v28-e90301-s001.doc]

**ARTICLE NO : 90301**

**ABSTRACT 408 WORDS**

The authors have provided proof of purchase for the figures containing identifiable individuals; Figures 1-6 therefore do not require any querying for consent, etc, and can be retained.

Google’s Display & Video 360 (DV360) platform

community-based organizations (CBOs)

click-through rate (CTR), and cost metrics cost per click (CPC), cost per thousand impressions (CPM), and cost per view (CPV)

Community-Engagement Research Alliance

Boat People SOS-Houston (BPSOSH)

(Consolidated Standards of Reporting Trials of Electronic and Mobile HEalth Applications and onLine TeleHealth) CONSORT-EHEALTH

Google Analytics (GA)

Health Belief Model (HBM)

Protection Motivation Theory (PMT)

Elaboration Likelihood Model (ELM)

[Analysis of Variance] ANOVA

US Department of Health and Human Services (HHS)

large language models (LLM)

machine translation (MT)
